# α Phase-Amplitude Tradeoffs Predict Visual Perception

**DOI:** 10.1523/ENEURO.0244-21.2022

**Published:** 2022-02-22

**Authors:** Camille Fakche, Rufin VanRullen, Philippe Marque, Laura Dugué

**Affiliations:** 1 Université de Paris, Integrative Neuroscience and Cognition Center Unité Mixte de Recherche 8002, Centre National de la Recherche Scientifique, Paris F-75006, France; 2 Centre National de la Recherche Scientifique, CerCo, Centre de Recherche Cerveau et Cognition, Unité Mixte de Recherche 5549, Université de Toulouse, Toulouse F-31052, France; 3 Toulouse NeuroImaging Center, Unité Mixte de Recherche 1214, INSERM Institut National de la Santé et de la Recherche Médicale, Toulouse F-31024, France; 4 Médecine Physique et de Réadaptation, Centre Hospitalier Universitaire Rangueil, Toulouse F-31059, France; 5 Institut Universitaire de France (IUF), Paris F-75005, France

**Keywords:** α oscillations, cortical excitability, EEG, phase-amplitude tradeoffs, TMS, visual perception

## Abstract

Spontaneous α oscillations (∼10 Hz) have been associated with various cognitive functions, including perception. Their phase and amplitude independently predict cortical excitability and subsequent perceptual performance. However, the causal role of α phase-amplitude tradeoffs on visual perception remains ill-defined. We aimed to fill this gap and tested two clear predictions from the pulsed inhibition theory according to which α oscillations are associated with periodic functional inhibition. (1) High-α amplitude induces cortical inhibition at specific phases, associated with low perceptual performance, while at opposite phases, inhibition decreases (potentially increasing excitation) and perceptual performance increases. (2) Low-α amplitude is less susceptible to these phasic (periodic) pulses of inhibition, leading to overall higher perceptual performance. Here, cortical excitability was assessed in humans using phosphene (illusory) perception induced by single pulses of transcranial magnetic stimulation (TMS) applied over visual cortex at perceptual threshold, and its postpulse evoked activity recorded with simultaneous electroencephalography (EEG). We observed that prepulse α phase modulates the probability to perceive a phosphene, predominantly for high-α amplitude, with a nonoptimal phase for phosphene perception between –π/2 and –π/4. The prepulse nonoptimal phase further leads to an increase in postpulse-evoked activity [event-related potential (ERP)], in phosphene-perceived trials specifically. Together, these results show that α oscillations create periodic inhibitory moments when α amplitude is high, leading to periodic decrease of perceptual performance. This study provides strong causal evidence in favor of the pulsed inhibition theory.

## Visual Abstract

**Figure:**
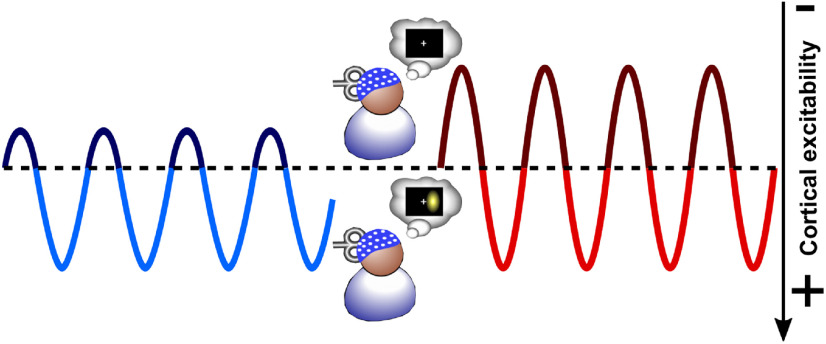

## Significance Statement

The pulsed inhibition theory predicts that the functional inhibition induced by high-α oscillations’ amplitude is periodic, with specific phases decreasing neural firing and perceptual performance. In turn, low-α oscillations’ amplitude is less susceptible to phasic moments of pulsed inhibition leading to overall higher perceptual performance. Using transcranial magnetic stimulation (TMS) with simultaneous electroencephalography (EEG) recordings in humans, we found that specific phases of spontaneous α oscillations (∼10 Hz) decrease cortical excitability and the subsequent perceptual outcomes predominantly when α amplitude is high. Our results provide strong causal evidence in favor of the pulsed inhibition theory.

## Introduction

α Brain oscillations (8–12 Hz) play a role in various cognitive functions, including visual perception (VanRullen, 2016a; Dugué and VanRullen, 2017; Clayton et al., 2018; Samaha et al., 2020; Kienitz et al., 2021), and are proposed to support active functional inhibition (Pfurtscheller and Lopes da Silva, 1999). Specifically, low parieto-occipital α amplitude recorded in human is correlated with a higher probability to perceive a near-threshold visual stimulus (Ergenoglu et al., 2004; Sauseng et al., 2005; Thut et al., 2006; van Dijk et al., 2008; Händel et al., 2011). Similarly, specific α phases lead to better detection performance while opposite phases lead to impaired performance (Varela et al., 1981; Busch et al., 2009; Mathewson et al., 2009; Dugué et al., 2011a; Samaha et al., 2015). Perception fluctuates periodically over time, along with the phase of α oscillations.

α Oscillations’ amplitude and phase further seem to predict cortical excitability. Spiking activity recorded in macaques is higher at the trough of the α cycle, and for lower α amplitude (Bollimunta et al., 2008; Haegens et al., 2011, 2015; van Kerkoerle et al., 2014). Moreover, functional magnetic resonance imaging studies have shown that the cortical blood oxygenation level-dependent response fluctuates along with the phase of α oscillations in visual cortex (V1/V2; Scheeringa et al., 2011), and increases when α amplitude decreases (Goldman et al., 2002; Moosmann et al., 2003). Critically, transcranial magnetic stimulation (TMS) studies went beyond such correlational evidence. When applied over V1/V2, single-pulse TMS can elicit phosphenes (illusory percepts) depending on cortical state, i.e., when cortical excitability is sufficiently high. Phosphene perception leads to a higher event-related potential (ERP) than the absence of percept (Taylor et al., 2010; Dugué et al., 2011a; Samaha et al., 2017). Interestingly, studies have shown that phosphene perception is higher for low prepulse α amplitude (Romei et al., 2008; Samaha et al., 2017), and fluctuates according to α phase (Dugué et al., 2011a; Samaha et al., 2017). These studies independently suggest that both α oscillations’ amplitude and phase modulate cortical excitability and causally predict visual perception. However, their joint causal effects are still ill-defined.

Here, we address this question in the framework of the pulsed inhibition theory (Klimesch et al., 2007; Jensen and Mazaheri, 2010; Mathewson et al., 2011), which makes two clear predictions regarding α phase-amplitude tradeoffs: (1) high-α amplitude exhibits states of cortical inhibition at specific phases associated with lower perceptual performance (nonoptimal phases); while (2) low-α amplitude is less susceptible to phasic pulsed inhibition, leading to high perceptual performance. A few studies have investigated the phase-amplitude tradeoffs (i.e., effect of the interaction between phase and amplitude) of low frequency oscillations on sensory perception and motor functions in human (Table 1). Although most of these studies observed a phase effect on task performance exclusively (or stronger) for high-α (or lower frequencies) amplitude (Mathewson et al., 2009; Ng et al., 2012; Ai and Ro, 2014; Bonnefond and Jensen, 2015; Herrmann et al., 2016; Spitzer et al., 2016; Kizuk and Mathewson, 2017; Hussain et al., 2019; Alexander et al., 2020), one found a phase effect for low-α amplitude exclusively (Busch and VanRullen, 2010), some found no difference between high-α and low-α amplitude (Milton and Pleydell-Pearce, 2016; Harris et al., 2018), and finally, some found no phase effect (Zoefel and Heil, 2013; Madsen et al., 2019). Importantly, most of these studies are correlational (Table 1). Only two (Hussain et al., 2019; Madsen et al., 2019) investigated the causal link between spontaneous α oscillations’ amplitude and phase, cortico-spinal excitability, assessed with TMS to evoke a motor-evoked potential (MEP) on the hand muscle, and the subsequent motor performance. Other studies found a causal phase effect of spontaneous α oscillations on cortico-spinal excitability and the associated MEP for high-α amplitude, but used the absence of α oscillations as control thus not comparing phase-effects between a high-α and low-α amplitude condition (Schaworonkow et al., 2018, 2019; Stefanou et al., 2018; Zrenner et al., 2018; Bergmann et al., 2019).

**Table 1:** Electro/magneto-encephalography (EEG/MEG) experiments investigating oscillations phase-amplitude tradeoffs on behavioral performance in human

Study	Method	Frequency band	Frequency	Oscillatory activity	Modality	Behavioral measure	Main results
Studies using TMS
Visual modality
Current study	EEG	α	10 Hz	Spontaneous	Visual	Near-threshold TMS-induced phosphene perception	Phase-amplitude effect on detection, stronger for high amplitude
Other modalities
Hussain et al. (2019)	EEG	α, β	8–12 Hz, 13–30 Hz	Spontaneous	Motor	Supra-threshold TMS-induced MEP	Optimal phase (leading to increased MEP) reverses between low-α and high-α amplitude; no phase-amplitude effect for β
Madsen et al. (2019)	EEG	α	7–13 Hz	Spontaneous	Motor	Supra-threshold TMS-induced MEP	No phase-amplitude effect on MEP amplitude
Studies not using TMS
Visual modality
Alexander et al. (2020)	EEG	α	5.6–14.4 Hz	Spontaneous	Visual	Detection of near-threshold stimulus through eyes closed	Phase effect on detection for high amplitude
Bonnefond and Jensen (2015)	MEG	α	9–12 Hz	Stimulus-locked (retention interval)	Visual	Sternberg working memory task with supra-threshold stimuli	Phase effect on γ power for high amplitude
Busch and VanRullen (2010)	EEG	θ	7 Hz	Spontaneous (amplitude attentionally modulated)	Visual	Detection of spatially attended or unattended near-threshold visual stimulus	Phase effect on detection and GFP for low amplitude
Harris et al. (2018)	EEG	θ, α	5 Hz, 11–15 Hz	Stimulus-locked (α amplitude attentionally modulated)	Visual	Detection of spatially attended or unattended near-threshold visual stimulus	No phase-amplitude effect on detection
Kizuk and Mathewson (2017)	EEG	α	12 Hz	Entrained	Visual	Detection of spatially attended or unattended metacontrast masked stimulus (75% performance)	Tendency for phase-amplitude effect on detection, stronger for high amplitude
Mathewson et al. (2009)	EEG	α	10 Hz	Stimulus-locked	Visual	Detection of metacontrast masked stimulus (70% performance)	Phase effect on detection for high amplitude
Milton and Pleydell-Pearce (2016)	EEG	α	7.8–12.7 Hz	Spontaneous (amplitude attentionally modulated)	Visual	Simultaneity judgement of spatially attended or unattended stimulus (50% performance)	No phase-amplitude effect on simultaneity judgement
Other modalities
Ai and Ro (2014)	EEG	α	8–12 Hz	Spontaneous	Somatosensory	Detection of near-threshold stimulus	Phase effect on detection for high amplitude
Herrmann et al. (2016)	MEG	δ	2 Hz	Entrained	Auditory	Detection of near-threshold stimulus	Phase effect on detection for high amplitude
Ng et al. (2012)	EEG	θ, α	2–6 Hz, 8–12 Hz	Entrained	Auditory	Detection of near-threshold stimulus	θ Phase-amplitude effect on detection, stronger for high amplitude; no phase-amplitude effect for α
Spitzer et al. (2016)	EEG	δ, θ, and α	3 Hz, 6.7 Hz, 11 Hz	Stimulus-locked	Visual, auditory, and somatosensory	Two-interval numerosity comparison task (80% performance)	δ Phase-amplitude effect on choice-predictive signals, stronger for high amplitude; no phase-amplitude effect for θ and α
Zoefel and Heil (2013)	EEG	δ	0.5 Hz	Entrained and spontaneous	Auditory	Detection of near-threshold stimulus	No phase-amplitude effect on detection neither for entrained nor for spontaneous oscillations

Note that studies independently investigating the phase and the amplitude of oscillations were not included in this table. We only selected studies specifically investigating the interaction between the instantaneous phase and the amplitude on behavioral performance. Entrained oscillations: oscillations with a nonrandom phase distribution induced by a repetitive stimulus presentation. Stimulus-locked oscillations: oscillations with a nonrandom phase distribution induced by a single stimulus. Spontaneous oscillations: oscillations with a random phase distribution. GFP, global field power; MEP, motor-evoked potential; TMS, transcranial magnetic stimulation.

We investigated the causal effect of spontaneous α phase-amplitude tradeoffs on cortical excitability and subsequent perceptual performance in the visual modality, and tested the predictions made by the pulsed inhibition theory using TMS and electroencephalography (EEG) in human. Cortical excitability was assessed using phosphene perception induced by single-pulse TMS applied over V1/V2 at perceptual threshold (∼50% detection), and its postpulse evoked activity. Our results validate both predictions, demonstrating that α oscillations create periodic inhibitory moments when α amplitude is high, leading to lower perceptual performance. Critically, both simulations and ERP analyses confirmed that the obtained results were not a mere analysis confound in which the quality of the phase estimation covaries with the amplitude.

## Materials and Methods

This study is a reappraisal of an early study from Dugué et al. (2011a), which focused on the causal link between the phase of ongoing α oscillations, cortical excitability, and visual perception. Here, we instead focused on the combined role of the phase and the amplitude of spontaneous α oscillations on the causal relation between cortical excitability and visual perception.

### Participants

As in the original study, the data from nine participants (eight male, 20–35 years old) were analyzed (for inclusion/exclusion criteria, see Dugué et al., 2011a). All participants fulfilled the standard inclusion criteria for TMS experiment (Rossi et al., 2009), gave their written informed consent, and were compensated for their participation. Human participants were recruited in Toulouse, France. The study was approved by the local French ethics committee Sud-Ouest et Outre-Mer I (IRB #2009-A01087-50) and followed the Declaration of Helsinki.

### TMS apparatus and EEG recording

Participants seated in a dark room, 57 cm from a computer screen (36.5° × 27° of visual angle). Their head was maintained using a chinrest and a headrest. A 70-mm figure-of-eight coil was placed over the right occipital pole (V1/V2; ∼1 cm above the inion and ∼2 cm away from the midline). The handle of the coil was oriented vertically, with the handle of the coil positioned dorsally to the coil itself, resulting in a ventral to dorsal electric current in the brain tissue. Biphasic TMS pulses were applied with a Magstim Rapid^2^ stimulator of 3.5 Tesla (Magstim). EEG was acquired simultaneously with a 64-channels Active Two Biosemi system, with DC recording at a sampling rate of 1024 Hz. Additional electrodes, Common Mode Sense (CMS) and Driven Right Leg (DRL), were placed 2 cm under the eyes of the participants and were used as reference and ground, respectively, to minimize TMS-induced EEG artifact. Finally, horizontal, and vertical electro-oculograms were recorded using three additional electrodes placed around the eyes.

### Experimental procedure

#### Phosphene screening and titration

Participants were selected based on their ability to perceive TMS-induced phosphenes in the left visual field. A train of seven pulses at 20 Hz and 70% of the TMS machine output intensity, i.e., suprathreshold, was applied over the right occipital pole (i.e., V1/V2; Dugué et al., 2011a; see also Dugué et al., 2016, 2019; Lin et al., 2021) while participants kept fixating at a central fixation. 24% of the participants did not perceive any phosphene (four out of 17) and were thus excluded from the main experiment. For each remaining participant, an individual phosphene perception threshold was determined by applying single pulses of TMS at varying intensities and asking them to report (with their dominant hand) whether they perceived a phosphene or not (left or right arrow on the computer keyboard, respectively).

#### Experimental session

**Figure 1.**
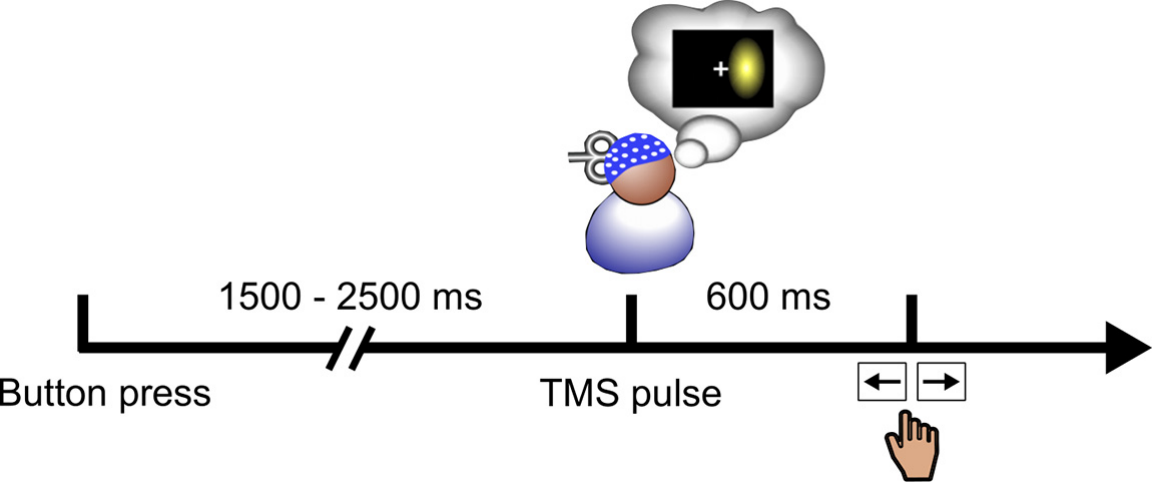
Experimental paradigm. Participants self-initiated the trial by pressing a button. After a random delay between 1500 and 2500 ms, a single (90% of trials) or a double (10% of trials) pulse of TMS was applied over V1/V2. After a 600-ms delay, participants indicated whether they perceived the phosphene or not with the left or right arrow, respectively.

Participants performed four blocks of 200 trials each, composed of 90% of test trials and 10% of catch trials (randomly interleaved). In the test trials, single-pulse TMS was applied at the perception threshold, on average across participants, phosphenes were perceived in 45.96 ± 7.68% of trials. In the catch trials, the stimulation intensity was kept the same, but instead of applying single pulses, double pulses (40-ms interval) were administered to monitor the validity of participants’ responses (91.66 ± 8.81% of phosphene perceived). In other words, participants were presumably not pressing the button randomly; the selected cortical location did in fact lead to phosphene perception when stimulated. Adding a second pulse of TMS with a short delay between the two pulses has a cumulative effect on neural activity, leading to suprathreshold stimulation (Ray et al., 1998; Gerwig et al., 2005; Kammer and Baumann, 2010). The longer the delay between the two pulses, the most likely such cumulative effect disappears, which in the present case would lead for the participants to perceiving two pulses. Debriefing with each participant confirmed that this was never the case. Throughout the experiment, participants kept fixating a central dot and pressed a button to start the trial (Fig. 1). The delay between the button-press and the subsequent TMS pulse varied randomly between 1500 and 2500 ms. After a 600-ms delay following the pulse, a response screen was displayed instructing the participants to indicate whether they perceived a phosphene or not with the left or right arrow, respectively. The percentage of phosphene perceived was monitored every 20 trials. When above 75% or below 25% of phosphene perceived in the test trials, the experimenter repeated the threshold procedure to select an intensity so as to maintain a threshold around 50% of phosphene perceived.

### EEG analyses

EEG analyses were performed with EEGLAB 13.6.5 (Swartz Center for Computational Neuroscience, University of California San Diego, California; Delorme and Makeig, 2004) and custom software written in MATLAB R2014b (The MathWorks).

#### EEG preprocessing

EEG data for test trials and channel localizations were imported into EEGLAB. EEG data were downsampled to 512 Hz, re-referenced to average reference, and epoched from −1500 to 1000 ms around the single-pulse of TMS. To minimize the artifact induced by the pulse, EEG data from −1 to 150 ms around the pulse was erased and replaced with a linear interpolation of the window boundaries. Epochs were finally manually inspected and rejected if artifacts (e.g., blinks) were detected. No electrode was rejected from the analysis.

#### Time-frequency decomposition

A time-frequency transform (morlet wavelets) was computed on single trials with the timefreq function from EEGLAB. The “cycles” parameter was set to [1, 15], the length of the filter increasing logarithmically from 1 to 15 cycles. The “freqs” parameter was set to [2, 100], producing frequencies that increase from 2 to 100 Hz.

#### Trial sorting by bin of amplitude

We then sorted the trials according to the amplitude of prepulse, spontaneous, α oscillations. The amplitude at each time-frequency point and for each trial, participant, and electrode was thus calculated. It corresponds to the absolute of the complex vector obtained from the time-frequency decomposition. For each trial and participant, we then averaged the amplitude across all electrodes (we had no a priori hypothesis regarding the electrode location of the effect) and time-frequency points selected based on the previous publication (Dugué et al., 2011a), i.e., time-frequency points at which a significant effect of the prepulse phase on phosphene perception was observed (from −400 to −50 ms before the pulse to avoid postpulse contamination on prepulse activity, and from 7 to 17 Hz). Trials were then sorted in three equally sized bins of low-amplitude, medium-amplitude, and high-amplitude trials. The percentage of phosphene perceived was computed for each bin of amplitude. Kruskal–Wallis test was used to test for significant difference in phosphene perception across bins. The next analyses were performed on the two extreme bins, i.e., low and high amplitude, exclusively, to clearly separate the low-α and high-α amplitude trials (with no possibility of overlap) using the least number of bins necessary to maximize the number of trials per condition. There were on average across the nine participants 108 ± 17.92 trials for the perceived-phosphene and 115.56 ± 15.86 trials for the unperceived-phosphene condition for the low-α amplitude trials, and 98.56 ± 30 trials for the perceived-phosphene and 126.11 ± 29.73 trials for the unperceived-phosphene condition for the high-α amplitude trials.

#### Phase-opposition

We calculated the phase-locking values (i.e., amount of phase concentration across trials), separately for low-α and high-α amplitude trials, and perceived-phosphene and unperceived-phosphene trials. The phase-locking value was obtained by, first, dividing the complex vectors obtained from the time-frequency decomposition by their length (i.e., instantaneous amplitude) thus normalizing for amplitude and keeping only the instantaneous phase (i.e., angle of the vectors). Second, the mean across trials of the normalized vectors was computed. The length of the average vector is now a measure of phase distribution, i.e., phase-locking across trials. For each amplitude condition, we subsampled the number of trials in the phosphene condition with the most trials to match the phosphene condition with the least trials. This subsampling procedure was repeated 100 times with a different subset of selected trials, and then we averaged the iterations. For each participant, phase-locking values were then summed across perceived-phosphene and unperceived-phosphene trials to obtain phase-opposition sums (POSs). POSs were then averaged across all electrodes, separately for low-α and high-α amplitude trials.

This measure of phase opposition is designed to give a low value when summing over two conditions both with uniform (random) phase distributions. On the other hand, POS is high when summing over two conditions both with strong phase-locked distributions across trials, as would happen when two conditions are associated with opposite phases. Since POS is computed on spontaneous (prepulse) activity, i.e., the phase distribution across all trials can be assumed to be uniform (see below for further statistics), if the phase is locked across trials for one specific condition (half of the overall trials) then the phase of the other condition (other half of the overall trials) would logically be locked in the opposite direction, leading to a high POS value.

This average was then compared with a surrogate distribution obtained with a permutation procedure (Dugué et al., 2011a, 2015; VanRullen, 2016b) consisting in shuffling the perceived-phosphene and unperceived-phosphene labels (5000 repetitions) and recalculating phase-locking values (including subsampling) to obtain a surrogate phase-locking distribution under the null hypothesis that both perceived-phosphene and unperceived-phosphene trials have a uniform phase distribution, and further summed across the two surrogate distributions to compute a surrogate POS, characterized by a given mean and SEM (Standard Error of the Mean; separate procedure for low-α and high-α amplitude). *Z*-scores were computed by comparing the experimentally obtained POS to the mean and SEM of the surrogate POS: *Z*-scores = (POS – surrogate POS mean)/surrogate POS SEM.

False discovery rate (FDR) correction for multiple comparisons was further applied to *p*-values. A topographical analysis on the *z*-scores revealed two regions of interest (ROIs) involved in the phase-opposition effect (occipital and frontal). We repeated the previous analyses for these regions.

Specific time-frequency points were then selected for further analyses. We selected the time points separately for occipital and frontal electrodes/ROIs for which Dugué et al. (2011a) observed the maximal phase effect between perceived-phosphene and unperceived-phosphene conditions at electrodes PO3 (in occipital ROI: −77 ms) and AFz (in frontal ROI: −40 ms), respectively. We selected the 10.7-Hz frequency for both occipital and frontal electrodes/ROIs based on the fast Fourier transform (FFT) performed on prepulse ERPs (see below), this frequency is identical to the frequency obtained when performing FFTs on prepulse EEG time series (see below, FFT on prepulse EEG time series). Note that the frequency resolution differs between the wavelet decomposition and the FFT. Thus, for all wavelet decomposition related analyses, we used the closest frequency (10.7 Hz) to the peak observed in the FFT analyses (10.24 Hz).

We further ensured, at these selected time-frequency points, that the prepulse phase-opposition effect was not because of a contamination by the wavelet decomposition from the postpulse activity, separately for electrode PO3 and AFz. We thus tested whether the phase was uniformly distributed for both low-α and high-α amplitude trials. For each trial, phases were extracted and averaged across participants, separately for perceived-phosphene and unperceived-phosphene conditions, and the uniformity of the distribution across all trials was tested with a Rayleigh test from the Circular Statistics Toolbox (P. Berens, CircStat: A MATLAB Toolbox for Circular Statistics, Journal of Statistical Software, Volume 31, Issue 10, 2009 http://www.jstatsoft.org/v31/i10, Berens, 2009). For both amplitude conditions, and for both electrode PO3 (low-α amplitude trials: *p* = 0.081657, κ = 0.1883; high-α amplitude trials: *p* = 0.22 314, κ = 0.13 517) and electrode AFz (low-α amplitude trials, *p* = 0.65 929, κ = 0.076575; high-α amplitude trials, *p* = 0.19 943, κ = 0.14 016), the tests did not reveal a significant effect suggesting that the phase was uniformly distributed across trials.

Finally, *post hoc* one-tailed *t* tests were performed to investigate whether POS, computed for each participant at the selected time-frequency points and averaged across electrodes for the occipital and the frontal ROI separately, differed significantly between low-α and high-α amplitude trials. This analysis was similarly performed for several versions of α amplitude binning (i.e., 2, 3, 4, and 5). On average across the nine participants, phosphene-conditions, and α-amplitude conditions, there were 167.94 ± 36.01 trials per bin in the two-bin version, 111.96 ± 24.29 trials in the three-bin version, 83.97 ± 18.41 trials in the four-bin version, and 67.18 ± 15.39 trials in the five-bin version.

#### FFT on prepulse EEG time series

To confirm further that the high-α as well as the low-α amplitude conditions both contained α oscillations, EEG time series from −600 to −1 ms were analyzed with an FFT (500 points zero padding), independently for the occipital and frontal ROI, for each α-amplitude condition, trial and participant. The resulting amplitude spectra were then averaged across trials and participants and plotted from 2 to 40 Hz. One-tailed *t* tests were used to compare individual 10.24-Hz peaks to their corresponding 1/f aperiodic component. The 10.24-Hz peaks were further compared between low-α and high-α amplitude conditions with one-tailed *t* tests. To ensure that the significant difference between the two conditions did not come from a difference in their aperiodic 1/f component, we also fitted the amplitude spectra to the 1/f component for each participant and compared the 1/f component at 10.24 Hz between low-α and high-α amplitude conditions with two-tailed *t* tests.

#### Simulations

**Figure 2.**
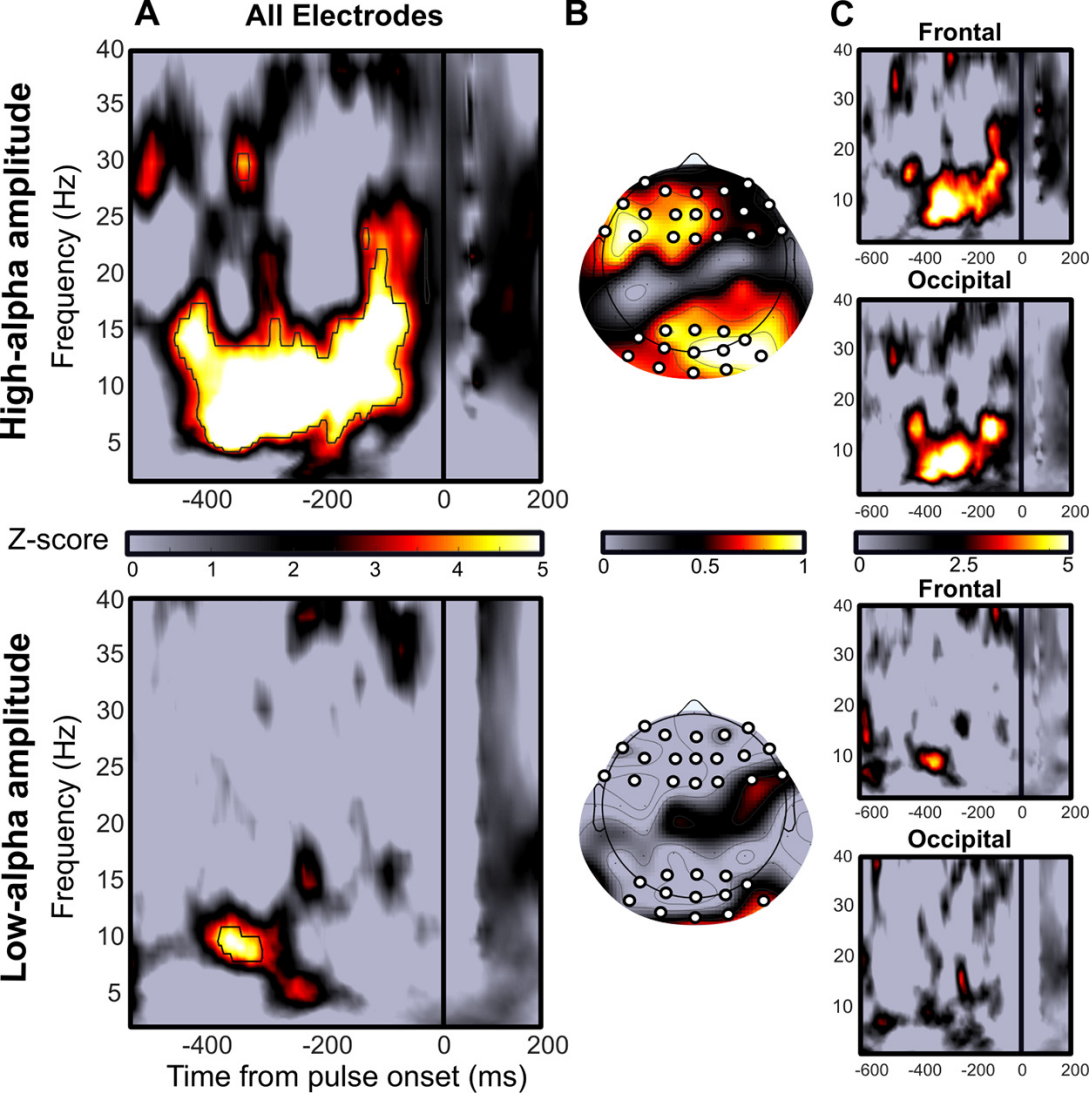
The phase of spontaneous α oscillations predicts phosphene perception mainly for high-α amplitude. This figure is supported by Extended Data Figures 2-1, 2-2, 2-3. Upper panel, Phase-opposition computed, respectively, on high-α amplitude trials. Lower panel, Low-α amplitude trials. ***A***, *Z*-scores map of phase-opposition between perceived-phosphene and unperceived-phosphene conditions averaged across nine participants and all 64 electrodes. Colormap, *Z*-scores. Black outline, significant phase-opposition FDR corrected for multiple comparisons (FDR = 0.01, corresponding to *p*-values threshold of 2.08 × 10^–5^ for low-α amplitude condition, and 1.96 × 10^–5^ for high-α amplitude condition). There is a significant phase-opposition from −400 to −50 ms before the pulse, between 5 and 18 Hz when α amplitude is high. The effect is less extended across time and frequencies when α amplitude is low. ***B***, *Z*-scores topographies averaged across the time-frequency window identified in panel ***A*** for high-α amplitude. The effect is maximal in a frontal and an occipital ROI when α amplitude is high. The topography is less clear when α amplitude is low. White dots, electrodes of interest within each ROI. ***C***, *Z*-scores maps of phase-opposition computed separately for the frontal ROI (upper panel) and the occipital ROI (lower panel).

The phase-opposition analysis between low-α and high-α amplitude trials could be because of an analysis confound, i.e., with decreasing amplitude, the robustness of the phase estimation decreases. Hence, a control procedure ensured that the phase estimation was not impacted by amplitude covariation, especially relevant when interpreting phase-opposition in low-α amplitude trials. A time-frequency decomposition and phase-opposition analysis, identical to the one described above (Fig. 2), was performed on a simulated dataset. Four electrophysiological datasets were simulated with similar properties as those observed in our empirical data: one for each experimental condition (300 trials each), i.e., perceived-phosphene and unperceived-phosphene for low-α and high-α amplitude conditions. Specifically, each trial was created as a sum of sine waves from 2 to 40 Hz. The amplitude of the simulated signal was determined based on the empirical data. For frequencies from 2 to 7 and 13 to 40 Hz, an amplitude of 100 arbitrary units (au) was chosen. For frequencies from 8 to 12 Hz, amplitudes varied depending on the simulated condition. For the low-α amplitude condition, we selected amplitudes of 280 au and 320 au for perceived-phosphene and unperceived-phosphene condition, respectively. For the high-α amplitude condition, we selected amplitudes of 580 and 620 au for perceived-phosphene and unperceived-phosphene conditions, respectively. A random phase between 0 and 2π was selected for frequencies from 2 to 7 and 13 to 40 Hz. For frequencies from 8 to 12 Hz, the phase varied depending on the simulated condition, i.e., [0 π] for perceived-phosphene for both low-α and high-α amplitude conditions, and [π 2π] for unperceived-phosphene for both low-α and high-α amplitude conditions. This phase distribution was applied to 80% of trials. In the other 20% of trials, a random phase between 0 and 2π was selected. Finally, white noise (VanRullen, 2016b) was added to all simulated datasets (amplitude: 4000 au) to match the empirically observed averaged *z*-score of phase-opposition (Fig. 2).

10.1523/ENEURO.0244-21.2022.f2-1 Extended Data Figure 2-1 The phase effect on phosphene perception is higher for high-α compared to low-α amplitude trials. POS computed for several binning versions of α amplitude trials, at 10.7 Hz and (***A–D***) at –77 ms prepulse, and averaged across electrodes within the occipital ROI, (***E–H***) at –40 ms prepulse, and averaged across the electrodes within the frontal ROI. Dots, POS for individual participants. Circles, POS averaged across the nine participants. All following analyses were performed on the three-bin condition (***B***, ***F***), i.e., trials were binned in low-α, medium-α (med), and high-α amplitude trials, discarding the medium-α amplitude bin. Download Figure 2-1, TIF file.

10.1523/ENEURO.0244-21.2022.f2-2 Extended Data Figure 2-2 Both low-α and high-α amplitude oscillations simulated datasets show a similar phase effect on perception. Left panel, Phase-opposition computed on simulated low-α amplitude trials. Right panel, Simulated high-α amplitude trials. *Z*-scores maps of phase-opposition between perceived-phosphene and unperceived-phosphene conditions. Colormap, *Z*-scores. Between low-α and high-α amplitude simulated trials, there is a comparable phase-opposition between perceived-phosphene and unperceived-phosphene conditions, from 5 to 18 Hz. Download Figure 2-2, TIF file.

**Figure 3.**
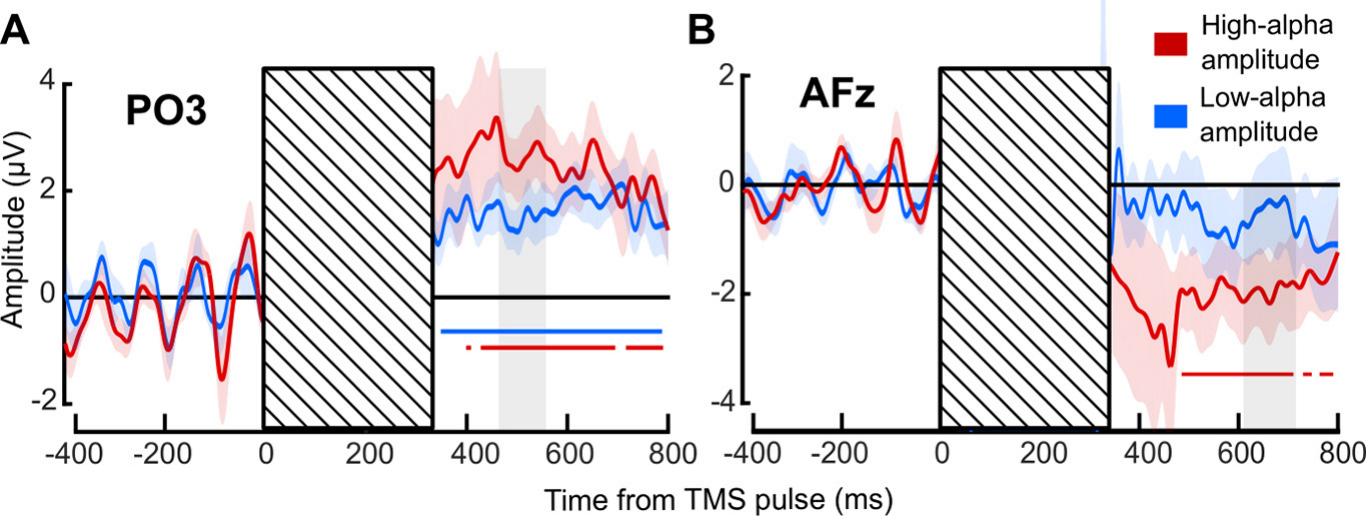
Difference between perceived-phosphene ERP and unperceived-phosphene ERP. This figure is supported by Extended Data Figure 3-1. ***A***, ERP difference at electrode PO3 between perceived-phosphene and unperceived-phosphene conditions averaged across nine participants. ***B***, ERP difference at electrode AFz. Red, high-α amplitude trials; blue, low-α amplitude trials. Colored shaded areas, SEM. Striped areas, mask the TMS-induced artifact. Colored solid horizontal lines, significant ERP difference against zero (significant cluster for PO3, low-α and high-α amplitude and AFz, high-α amplitude; *p* < 0.001). Gray shaded areas, selected time window of interest.

10.1523/ENEURO.0244-21.2022.f2-3 Extended Data Figure 2-3 Prepulse oscillatory activity in the α frequency band in both low-α and high-α amplitude conditions. Amplitude spectra computed on the EEG time-series from –600 to –1 ms relative to pulse onset, for the occipital ROI (left panel) and the frontal (right panel) ROI. Red color, high-α amplitude condition; blue color, low-α amplitude condition. Colored solid lines, amplitude spectra averaged across the nine participants between 2 and 40 Hz. Colored shaded areas, SEM. *, significant difference at 10.24 Hz between low-α and high-α amplitude conditions. Download Figure 2-3, TIF file.

##### ERPs

Previously preprocessed EEG data were further cleaned from the power line noise by applying a notch filter at 50 Hz (band-stop at 47–53 Hz) before epoching. ERPs, centered on the pulse onset, were computed as the average of trials for each α-amplitude condition, phosphene-perception condition, and participant. The difference of ERPs between perceived-phosphene and unperceived-phosphene conditions was computed, separately for low-α and high-α amplitude conditions. The ERP differences were then compared against zero with repeated measures one-tailed *t* tests (from 350 to 800 ms). Correction for multiple comparisons was applied following a cluster procedure. For each participant, surrogate ERPs were obtained by shuffling the perceived and unperceived phosphene labels (500 repetitions). *t* tests were recomputed similarly as before, and the number of consecutive significant time points (surrogate cluster size) for each repetition was stored. The *p*-value for each empirical cluster was then computed as the proportion of surrogate clusters that were larger than the empirical cluster. Using an α level of 0.05, we considered an empirical cluster significant if its size was larger than at least 95% of the surrogate clusters.

#### FFT on prepulse ERPs

The use of TMS to induce phosphene perception rather than an external stimulation allows for direct access to the instantaneous state of the spontaneous brain oscillations. In other words, one can directly compute the prepulse ERP differences (time before pulse onset) between perceived-phosphene and unperceived-phosphene conditions, to assess spontaneous oscillatory activity. In this case, when comparing ERP differences between perceived-phosphene and unperceived-phosphene conditions, separately for low-α and high-α (presorted) amplitude trials, there is no analysis confound coming from a less accurate phase estimation when α amplitude is low. ERP differences from −400 to 0 ms were then analyzed with an FFT (500 points zero padding), independently for electrodes PO3 and AFz, for each α-amplitude condition and for each participant. The resulting amplitude spectra were then averaged across participants and plotted from 2 to 40 Hz (a peak at 10.24 Hz was observed for both electrodes and for each α-amplitude condition). We then performed the following steps to test for a difference between high-α and low-α amplitude trials in the resulting amplitude spectra: (1) difference of the averaged amplitude spectra between high-α and low-α amplitude trials; (2) fit of this difference to the 1/f component; (3) removing of the 1/f component; (4) Gaussian fit of the resulting amplitude spectra to extract the frequency window showing a difference between high-α and low-α amplitude trials; (5) statistical comparison of the amplitude spectra (uncorrected for 1/f) between high-α and low-α amplitude trials conditions with a one-tailed *t* test. To ensure that the obtained significant difference between low-α and high-α amplitude conditions was not because of a difference in their 1/f aperiodic components, we fitted the amplitude spectra to their 1/f component and computed the area under the curve, for each participant, separately for low-α and high-α amplitude conditions, for both electrodes PO3 and AFz. The area under the curve of the low-α amplitude condition was compared with the one of the high-α amplitude condition with a two-tailed *t* test.

Finally, an FFT (500 points zero padding) was performed separately for the ERP of perceived-phosphene and unperceived-phosphene conditions on each participant. We then computed phase-locking values across participants to assess the interindividual variability at 10.24 Hz for low-α and high-α amplitude trials separately (frequency at which a peak of amplitude was observed in the amplitude spectra of the ERP difference between phosphene perceived and unperceived conditions). The sum of phase-locking values across participants of perceived-phosphene and unperceived-phosphene conditions was computed, for low-α and high-α amplitude trials separately, and evaluated statistically with a permutation procedure. *P*-values were estimated by comparing these POSs to the mean and SEM of the surrogate distribution obtained by shuffling the perceived-phosphene and unperceived-phosphene labels (repeated 500 times), for low-α and high-α amplitude trials separately, before replicating the previous analysis on the surrogate ERPs.

#### FFT on postpulse ERPs

As mentioned above, the advantage of the present TMS procedure is that it allows for direct access to the instantaneous state of the spontaneous brain oscillations. In other words, prepulse spontaneous activity is readily observable on the ERP. To ensure that postpulse ERPs were not contaminated by spontaneous α oscillations (i.e., that the TMS pulse here reset α oscillations), an FFT (500 points zero padding) was performed on the ERPs of the perceived-phosphene and unperceived-phosphene conditions, from 400 to 800 ms, for electrodes PO3 and AFz separately, and for each α-amplitude condition and participant. One-tailed *t* tests against the aperiodic 1/f activity were used to test the significance of the 10.24-Hz peak.

#### Perceptual performance as a function of prepulse phase

Low-α and high-α amplitude trials were sorted in nine phase bins at the selected time-frequency points (see above, Phase-opposition), separately for the occipital ROI, as well as the specific electrode PO3 (−77 ms, 10.7 Hz), and the frontal ROI, as well as the specific electrode AFz (−40 ms, 10.7 Hz). The percentage of perceived-phosphene was computed for each phase bin, α-amplitude condition, electrode, and participant, and further averaged across participants and electrodes. The values were finally normalized by dividing the percentage of perceived-phosphene averaged across phase bins, separately for each α-amplitude condition. A two-way repeated-measures ANOVA was performed to test for the main effect of phase bin. The percentage of variance explained was computed as the difference between the optimal phase (maximum percentage of phosphene perceived) and the opposite one.

#### ERP amplitude as a function of prepulse phase

Bin sorting was applied as described in the previous section. Then, the ERP difference between perceived-phosphene and unperceived-phosphene conditions for each phase bin and α-amplitude condition was computed. The maximum perceived-unperceived ERP difference was selected in the time window in which an ERP difference between low-α and high-α amplitude was detected, according to repeated measures two-tailed *t* test for each time point from 350 to 800 ms (PO3: from 482 to 513 ms; AFz: from 605 to 728 ms). A two-way repeated-measures ANOVA was performed to test for a main effect of phase bin and interaction between phase bin and α-amplitude (results regarding the main effect of α-amplitude were not interpreted). Specifically, we tested the hypotheses that (1) the ERP difference between perceived-phosphene and unperceived-phosphene conditions depended on the phase of spontaneous α oscillation, and (2) that this phase effect is stronger for high-α amplitude trials. Finally, we calculated the maximum ERP amplitude of the perceived-phosphene and unperceived-phosphene conditions separately, independently for phase bin centered on –π/4 and π/2, corresponding, respectively, to the maximum and the minimum ERP difference, and low-α and high-α amplitude trials. For each α-amplitude condition, we fitted the data to a linear mixed-effect model with phase bins and phosphene conditions as fixed effects, and participants as random effects. *Post hoc* analyses were done with one-tailed *t* tests.

## Results

Single-pulse TMS was applied over the right occipital cortex (V1/V2) in nine healthy participants, at threshold intensity (45.96 ± 7.68% of phosphene perceived across participants) while simultaneously recording EEG. Previous analysis of this dataset (Dugué et al., 2011a) revealed that the phase of spontaneous α oscillations in the time-frequency window from −400 to −50 ms prepulse, and from 7 to 17 Hz, predicts the perceptual outcome. This phase effect explained ∼15% of the variability in phosphene perception. Here, trials were split according to low-amplitude, medium-amplitude, and high-amplitude of the prepulse spontaneous α oscillations (within the same time frequency-window as in Dugué et al., 2011a). We tested the two predictions made by the pulsed inhibition theory: (1) high-α amplitude induces periodic inhibitory moments leading to poor perceptual performance; and (2) low-α amplitude is less susceptible to phasic inhibition, and lead to overall higher perceptual performance. We first analyzed phosphene detection for each α-amplitude condition. We observed that phosphene detection rate depends on α amplitude with the highest detection being in low-α (48.22 ± 4.88%) then medium-α (45.94 ± 7.92%), and high-α (43.75 ± 11.57%) amplitude trials (Kruskal–Wallis: *p* = 0.0553, Cohen’s *d* (effect size) = 0.88). Note that the earlier study (Dugué et al., 2011a) was not optimized to test the predictions made by the pulsed inhibition theory. However, the present results argue in its favor, with higher phosphene perception when α amplitude is low (Romei et al., 2008). In the next analyses, we discarded the medium-α amplitude trials to concentrate on the low-α and high-α amplitude conditions. This allowed us to clearly separate the two types of amplitude trials, while maximizing the number of trials per condition (see also Extended Data Fig. 2-1 for further assessment of such amplitude binning procedure).

To investigate the potential joint effect of the amplitude and the phase of spontaneous α oscillations on phosphene perception, we calculated POS (see Materials and Methods), separately for low-α and high-α amplitude conditions. Specifically, this analysis assesses whether phosphene perception is modulated by significantly different phases of the α cycle by computing the sum of phase-locking values (i.e., the amount of phase concentration across trials) over the perceived-phosphene and unperceived-phosphene conditions (Dugué et al., 2011a,b, 2015; VanRullen, 2016b). Spontaneous activity is characterized by a uniform phase distribution across all trials. Thus, if the phase is locked across trials for the perceived-phosphene condition (approximately half of the overall trials) then the phase of the unperceived-phosphene condition (other half of the overall trials) will logically be locked in the opposite direction, leading to a strong POS. Conversely, a weak POS value can only be obtained if both perceived and unperceived-phosphene conditions have near-random phase distributions, i.e., if phase does not affect phosphene perception. For high-α amplitude (Fig. 2, top raw), this analysis revealed a strong phase-opposition between perceived-phosphene and unperceived-phosphene conditions across all participants and electrodes, from −400 to −50 ms prepulse, and in the frequency range from 5 to 18 Hz (Fig. 2*A*). This effect remained significant after FDR correction for multiple comparisons (FDR = 0.01, corresponding to a *z*-score threshold of 4.27, a *p*-value threshold of 1.96 × 10^−5^, and a Cohen’s *d* threshold approaching infinity). The corresponding topography revealed that the phase-opposition effect was maximal over occipital and frontal electrodes (Fig. 2*B*,*C*). The analysis was replicated on low-α amplitude trials (Fig. 2, bottom raw). The overall strength of the effect was less important, i.e., effect less extended across time and frequency, for low-α amplitude trials (remained significant after FDR correction, FDR = 0.01, corresponding to a *z*-score threshold of 4.26, a *p*-value threshold of 2.08 × 10^−5^, and a Cohen’s *d* approaching infinity), and showed a less informative topography of the effect. A *post hoc* analysis revealed that POS values, averaged across electrodes, separately for the occipital and frontal ROIs at the respective selected time-frequency points (see Materials and Methods), were significantly higher for high-α compared with low-α amplitude trials at (10.7 Hz, −77 ms) for the occipital ROI (one-tailed *t* test: *p* = 0.0016, Cohen’s *d* = 1.9645, CI = [0.042; infinity]; Extended Data Fig. 2-1*B*) and at (10.7 Hz, −40 ms) for the frontal ROI (one-tailed *t* test: *p* < 0.001, Cohen’s *d* = 2.1165, CI = [0.0457; infinity]; Extended Data Fig. 2-1*F*). Together, these results suggest that there is an optimal phase of spontaneous α oscillations that predicts phosphene perception. This phase effect is more robust across time and across frequencies and is significantly higher for high-α compared with low-α amplitude trials. Interestingly, Extended Data Fig. 2-1 further illustrates the impact of several binning versions of the previous analysis (from two to five bins). In all versions, there is a difference of POS between low-α and high-α amplitude trials (one-tailed *t* tests for all binning versions show *p*s < 0.004 and Cohen’s *d*s >1.27). However, increasing the number of bins decreases the number of trials in each bin. Consequently, all main analyses were performed on the three-bin version (excluding the middle bin) to clearly separate low-α and high-α amplitude trials while maximizing the number of trials per condition.

To ensure that the difference in phase effect observed between low-α and high-α amplitude trials does not depend on a poor estimation of the phase when α amplitude is low, we performed a control analysis based on simulations (Extended Data Fig. 2-2). The phase-opposition analysis displayed in Figure 2 was repeated on simulated data generated with the same parameters (amplitude ratio between low-α and high-α amplitude trials) than those observed in the empirical dataset (for more details, see Materials and Methods). The simulations show that in both the low-α and high-α amplitude conditions, POS between perceived-phosphene and unperceived-phosphene trials can be observed with similar time-frequency profiles. Thus, the effect observed in Figure 2 cannot be simply explained by an underpowered phase estimation in low-α amplitude trials but indeed reflects a functional neurophysiological brain process. In addition, we tested that α oscillations are actually present in prepulse, low-α amplitude trials (Extended Data Fig. 2-3). An FFT on the prepulse EEG activity revealed a peak at 10.24 Hz in the occipital ROI for both low-α (one-tailed *t* test against the 1/f aperiodic activity: *p* = 0.0354, Cohen’s *d* = 0.7371, CI = [11.7481; infinity]) and high-α (*p* = 0.0102, Cohen’s *d* = 0.9999, CI = [60.0927; infinity]) amplitude trials, and in the frontal ROI for both low-α (*p* = 0.0465, Cohen’s *d* = 0.6668, CI = [1.6258; infinity]) and high-α (*p* = 0.0162, Cohen’s *d* = 0.9527, CI = [29.2137; infinity]) amplitude trials. This analysis confirms that estimating the phase in low-α amplitude trials is indeed neurophysiologically relevant. Additionally, the peak at 10.24 Hz was significantly higher for high-α compared with low-α amplitude trials, for both the occipital (one-tailed *t* tests: *p* = 0.0268, Cohen’s *d* = 0.2176, CI = [6.8434; infinity]) and the frontal (*p* = 0.0316, Cohen’s *d* = 0.2485, CI = [4.1668; infinity]) ROI. This difference was unlikely because of a difference in the 1/f aperiodic activity between low-α and high-α amplitude conditions, i.e., there was no significant difference in the 1/f component at 10.24 Hz between low-α and high-α amplitude conditions for the occipital (two-tailed *t* tests: *p* = 0.2362; Cohen’s *d* = 0.1893, CI = [−9.8442; 34.4352]) and the frontal (*p* = 0.0867, Cohen’s *d* = 0.3361, CI = [−2.5463; 30.6516]) ROI.

**Figure 4.**
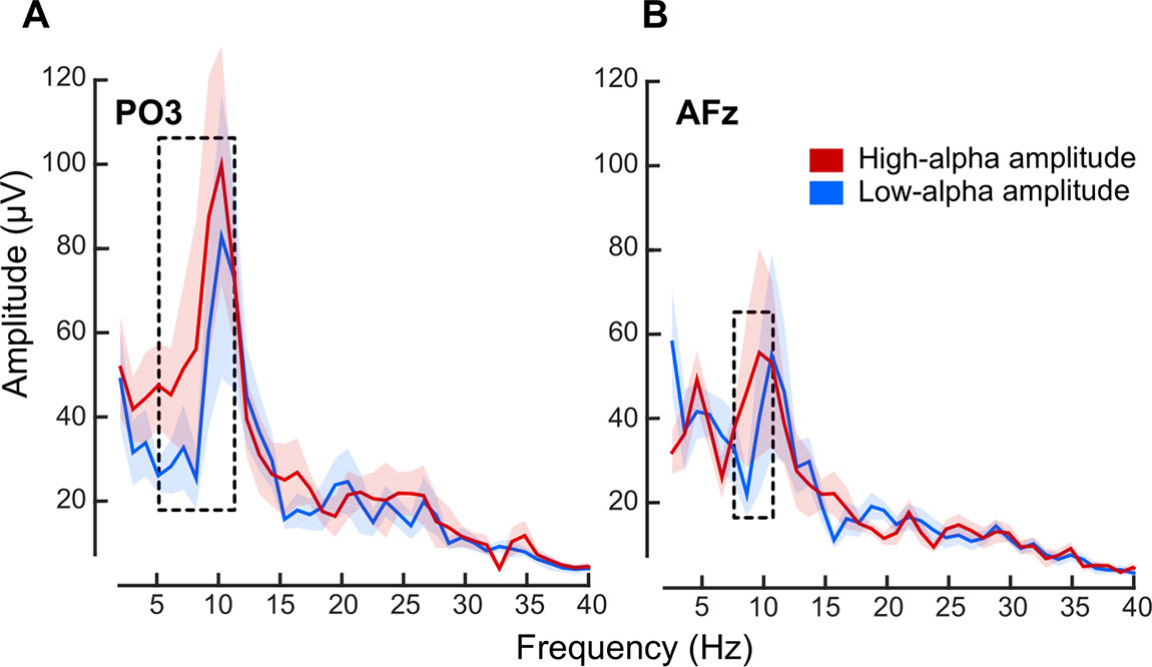
The prepulse ERP difference between perceived-phosphene and unperceived-phosphene conditions oscillates in α. ***A***, Frequency spectra computed on the ERP difference between perceived-phosphene and unperceived-phosphene conditions, on the prepulse period from −400 to 0 ms, respectively, for electrode PO3, and, ***B***, electrode AFz. Red color, high-α amplitude trials; blue color, low-α amplitude trials. Colored solid lines, frequency spectra averaged across the nine participants between 2 and 40 Hz. Shaded area, SEM. Dotted rectangle, significant difference between high-α and low-α amplitude trials averaged across frequency window from 5.12 to 11.26 Hz for electrode PO3, and from 7.16 to 10.24 Hz for electrode AFz (one-tailed *t* tests: *p* = 0.044 for PO3, *p* = 0.039 for AFz). Frequency peak at 10.24 Hz for both low-α and high-α amplitude for electrode PO3; at 10.24 Hz for low-α and 9.22 Hz for high-α amplitude for electrode AFz.

To further understand the link between prepulse spontaneous α oscillatory phase and amplitude, cortical excitability and phosphene perception, and address further a possible confound coming from a less accurate phase estimation when α amplitude is low (see Materials and Methods), we analyzed the ERP difference between perceived-phosphene and unperceived-phosphene conditions. Critically, the use of TMS to induce phosphene perception allows for direct access to the instantaneous state of the spontaneous brain oscillations. In other words, the prepulse ERP differences (time before pulse onset) between perceived-phosphene and unperceived-phosphene conditions, allows to assess spontaneous oscillatory activity. Thus, if there is an optimal phase for perception and an opposite, nonoptimal one, then for each participant the prepulse ERP for perceived-phosphene and for unperceived-phosphene should each oscillate in α, and so would the ERP difference. Additionally, if all participants share the same optimal phase, then the prepulse ERP difference averaged across participants should oscillate in α as well. We analyzed the ERP difference between perceived-phosphene and unperceived-phosphene conditions for electrodes PO3 and AFz (selected, respectively, in the occipital and frontal ROIs based on previous studies; Taylor et al., 2010; Dugué et al., 2011a; Fig. 3). For both electrodes and for both low-α and high-α amplitude trials, the ERP difference appeared periodic in the last 400 ms preceding the pulse (note that both low-α and high-α amplitude conditions show this effect). An FFT applied on the ERP difference of each participant in the prepulse period (−400–0 ms) showed a peak in amplitude for both electrode PO3 (10.24 Hz for both low-α and high-α amplitude; Fig. 4*A*) and electrode AFz (10.24 Hz for low-α and 9.22 Hz for high-α amplitude; Fig. 4*B*). Additionally, the amplitude of the prepulse oscillatory difference between perceived-phosphene and unperceived-phosphene was significantly higher for high-α amplitude compared with low-α amplitude trials, for the frequency window from 5.12 to 11.26 Hz for PO3 (one-tailed *t* test: *p* = 0.044, Cohen’s *d* = 0.361, CI = [0.813; infinity]; see Materials and Methods), and from 7.16 to 10.24 Hz for AFz (*p* = 0.039, Cohen’s *d* = 0.241, CI = [0.786; infinity]). This difference was unlikely because of a difference in the 1/f aperiodic activity between low-α and high-α amplitude conditions as their aperiodic activity did not differ significantly, neither for electrode PO3 (two-tailed *t* test: *p* = 0.063, Cohen’s *d* = 0.3131, CI = [−13.8069; 414.8763]) nor AFz (two-tailed *t* test: *p* = 0.806, Cohen’s *d* = 0.0264, CI = [−76.8006; 95.8066]). To further assess the interindividual variability, we calculated the sum of phase-locking values across participants at 10.24 Hz for perceived-phosphene and unperceived-phosphene conditions, separately for low-α and high-α amplitude trials, and for PO3 and AFz electrodes. In other words, we ask whether the prepulse ERP for each condition oscillates in-phase across all participants, and are in phase-opposition between perceived-phosphene and unperceived-phosphene conditions. We found that there is a phase-opposition between perceived-phosphene and unperceived-phosphene conditions for the electrode PO3, for high-α amplitude trials (permutation statistics: *z*-score = 1.9794, *p* = 0.0239, Cohen’s *d* = 1.756), but not for low-α amplitude trials (*z*-score = 0.9856, *p* = 0.1622, Cohen’s *d* = 0.6957), nor for AFz low-α (*z*-score = 0.2059, *p* = 0.4184, Cohen’s *d* = 0.1375) and high-α (*z*-score = 1.169, *p* = 0.1212, Cohen’s *d* = 0.8462) amplitude. Phosphene perception depends on an optimal phase of α oscillation at the occipital electrode PO3, when α amplitude is high. Together, these analyses suggest that phosphene perception alternates between optimal and nonoptimal phases of the α (10.24 Hz) oscillations in the 400-ms window before the pulse, with all participants sharing a similar optimal phase. This phase effect is predominant in the occipital region, and stronger when the α amplitude is high.

10.1523/ENEURO.0244-21.2022.f3-1 Extended Data Figure 3-1 ERPs for perceived-phosphene and unperceived-phosphene trials. ***A***, ERPs at electrode PO3 for perceived-phosphene and unperceived-phosphene trials averaged across the nine participants. ***B***, ERPs at electrode AFz. Red, high-α amplitude condition; blue, low-α amplitude condition. Light colors, perceived-phosphene condition; dark colors, unperceived-phosphene condition. Colored shaded areas, SEM. Striped area, mask the TMS-induced artifact. Download Figure 3-1, TIF file.

**Figure 5.**
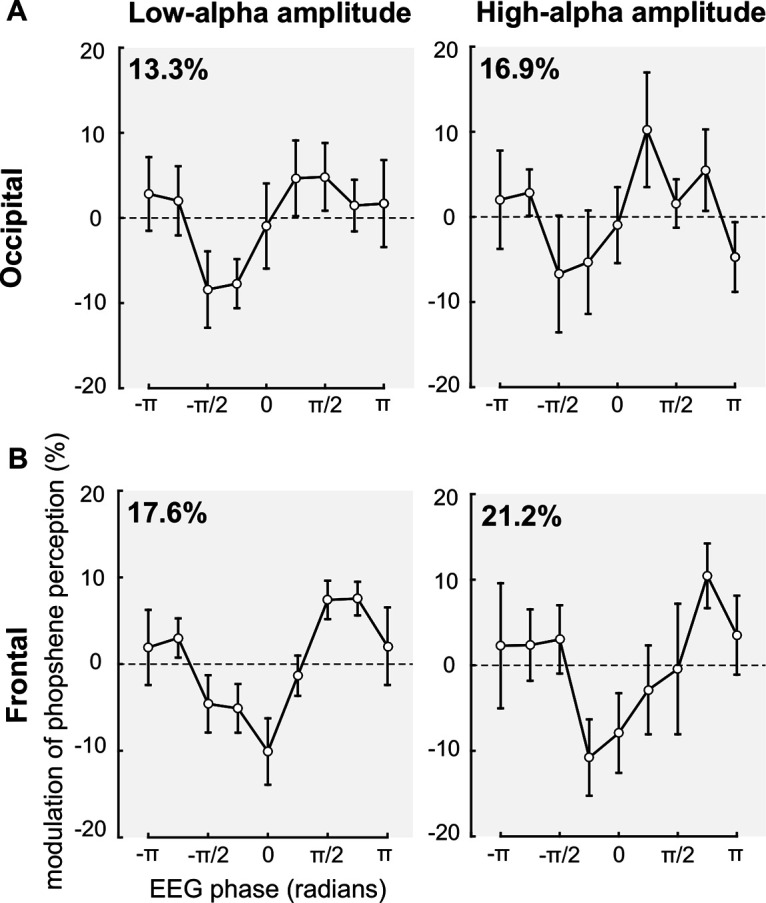
The phase π/2 of the α cycle is the optimal phase for phosphene perception. Left panels, Phosphene perception computed for nine phase bins (expressed in radians), normalized according to the average phosphene perception, and averaged across the nine participants and electrodes of interest, for low-α amplitude trials. Right panels, For high-α amplitude trials. Error bars, SEM. ***A***, Phosphene perception is plotted according to the instantaneous phase at −77 ms, 10.7 Hz, for the occipital ROI. Phosphene perception oscillates along with the α phase (two-way repeated-measures ANOVA: *F*_(1,8)_ = 2.117, *p* = 0.0467, η^2^ = 20.93), with an optimal phase for phosphene perception at the phase π/2 of the α cycle. The percentage of variance explained by the phase is more important for high-α (16.9% difference between π/2 and –π/2) compared with low-α (13.3%) amplitude trials. ***B***, Phosphene perception is plotted according to the instantaneous phase at −40 ms, 10.7 Hz for the frontal ROI. Phosphene perception oscillates along with the α phase (two-way repeated-measures ANOVA: *F*_(1,8)_ = 3.360, *p* = 0.0028, η^2^ = 29.58), with an optimal phase for phosphene perception at the phase π/2 of the α cycle. The percentage of variance explained by the phase is more important for high-α (21.2% difference between π/2 and –π/2) compared with low-α (17.6%) α amplitude trials.

Next, for each participant, we sorted low-α and high-α amplitude trials in nine bins according to the prepulse EEG phase, for the selected time-frequency points (occipital ROI: −77 ms, 10.7 Hz; frontal ROI: −40 ms, 10.7 Hz). Then, the percentage of phosphene perceived was calculated for each bin and averaged across participants (Fig. 5). A two-way repeated-measures ANOVA revealed a significant effect of the phase in both the occipital (*F*_(1,8)_ = 2.117, *p* = 0.0467, η^2^ (effect size) = 20.93, square sum (SS) = 0.345) and frontal (*F*_(1,8)_ = 3.360, *p* = 0.0028, η^2^ = 29.58, SS = 0.472) ROIs. There was no main effect of amplitude in either the occipital (*F*_(1,8)_ = 1.818, *p* = 0.2145, η^2^ = 18.51, SS = 0.001) or the frontal (*F*_(1,8)_ = 0.225, *p* = 0.6476, η^2^ = 2.74, SS < 0.001) ROIs, nor interaction in either the occipital (*F*_(1,8)_ = 0.249, *p* = 0.9794, SS = 0.048) or the frontal (*F*_(1,8)_ = 0.462, *p* = 0.8781, SS = 0.076) ROIs. Critically, the results show that the optimal phase for phosphene perception is centered on π/2 while the opposite phase, between –π/2 and –π/4, is nonoptimal. Finally, we observe that the percentage of variance explained by the phase is more important for high-α (occipital: 16.9% difference between π/2 and –π/2; frontal: 21.2%) compared with low-α (occipital: 13.3%; frontal: 17.6%) amplitude of spontaneous oscillations. We repeated this analysis for the individual electrodes PO3 and AFz and observed similar effects. A two-way repeated-measures ANOVA showed a significant effect of the phase for both electrodes PO3 (*F*_(1,8)_ = 2.113, *p* = 0.0472, η^2^ = 20.89, SS = 0.937) and AFz (*F*_(1,8)_ = 2.106, *p* = 0.0479, η^2^ = 20.84, SS = 0.908), no significant main effect of the amplitude for either electrode PO3 (*F*_(1,8)_ = 0.461, *p* = 0.5164, η^2^ = 5.45, SS = 0.002) or AFz (*F*_(1,8)_ = 0.002, *p* = 0.9633, η^2^ = 0.03, SS < 0.001), and no interaction for either electrode PO3 (*F*_(1,8)_ = 0.612, *p* = 0.7648, SS = 0.294) or AFz (*F*_(1,8)_ = 0.389, *p* = 0.9224, SS = 0.2).

To understand the role of spontaneous α oscillations phase-amplitude tradeoffs on cortical excitability and subsequent perceptual performance, we then focused on the postpulse evoked activity. Dugué et al. (2011a) previously observed a larger postpulse ERP in the perceived- than in the unperceived-phosphene trials, with a positive differential activity for PO3, and negative for AFz, between ∼300 and ∼600 ms. They interpreted these results as a physiological consequence of phosphene perception. Here, we computed the ERP difference between perceived-phosphene and unperceived-phosphene conditions, separately for low-α and high-α amplitude trials and observed a similar effect in both low-α and high-α amplitude conditions (Fig. 3; see also Extended Data Fig. 3-1 for ERPs on each condition separately). An FFT was computed from 400 to 800 ms after the pulse on the perceived-phosphene and unperceived phosphene ERP, separately for electrodes PO3 and AFz, and for low-α and high-α amplitude trials. There was no significant frequency peak at 10.24 Hz in the postpulse ERP amplitude spectra, in any of the conditions for both electrode PO3 (one-tailed *t* tests against the 1/f aperiodic component: low-α amplitude, perceived: *p* = 0.9244, Cohen’s *d* = −0.4967, CI = [−28.6420; infinity]; unperceived: *p* = 0.6837, Cohen’s *d* = −0.1176, CI = [−11.6204; infinity]; high-α amplitude, perceived: *p* = 0.4279, Cohen’s *d* = 0.0643, CI = [−17.1197; infinity]; unperceived: *p* = 0.0971, Cohen’s *d* = 0.3345, CI = [−4.1354; infinity]) and AFz (low-α amplitude, perceived: *p* = 0.9864, Cohen’s *d* = −1.3376, CI = [−36.8469; infinity]; unperceived: *p* = 0.5615, Cohen’s *d* = −0.0520, CI = [−15.0938; infinity]; high-α amplitude, perceived: *p* = 0.3038, Cohen’s *d* = 0.0322, CI = [−28.0728; infinity]; unperceived: *p* = 0.1811, Cohen’s *d* = 0.0610, CI = [−17.3280; infinity]). Thus, the postpulse signal likely does not contain sufficient prepulse information to translate into a contamination of the postpulse ERP.

**Figure 6.**
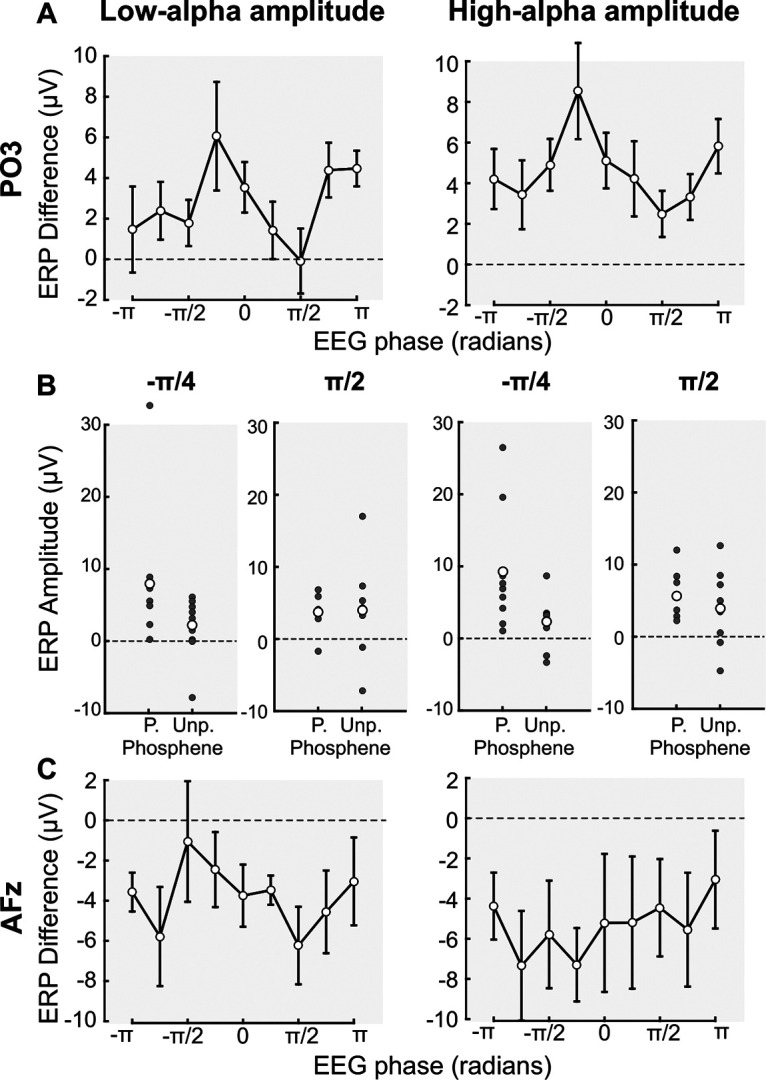
The ERP is higher for perceived-phosphene trials at the nonoptimal phase for phosphene perception, for the electrode PO3. Left panels, ERP difference between perceived-phosphene and unperceived-phosphene conditions computed for nine phase bins, and averaged across the nine participants, for low-α amplitude trials. Right panels, For high-α amplitude trials. ***A***, Single trials were sorted into nine phase bins according to the instantaneous phase at −77 ms, 10.7 Hz, for the electrode PO3. For each phase bin, the maximum perceived-unperceived ERP difference was computed. Errors bars, SEM. The ERP difference oscillates along with the α phase (*F*_(1,8)_ = 2.338, *p* = 0.0286, η^2^ = 22.62). The ERP difference was higher at the phase –π/4. ***B***, ERP for the perceived-phosphene and unperceived-phosphene conditions, for the electrode PO3, for the phase –π/4 and π/2. P., perceived-; Unp., unperceived-phosphene conditions. Gray dots, maximum ERP for each participant; white dots, averaged ERP across the nine participants. The maximum ERP at the phase –π/4 for perceived-phosphene trials was significantly higher compared with unperceived-phosphene trials at the phase –π/4 for both low-α (one-tailed *t* test, *p* = 0.0265) and high-α (*p* = 0.0074) amplitude trials type, and compared with unperceived-phosphene trials at the phase π/2 for both low-α (*p* = 0.0254) and high-α (*p* = 0.0253) amplitude trials type. ***C***, Single trials were sorted into nine phase bins according to the instantaneous phase at −40 ms, 10.7 Hz, for the electrode AFz. For each phase bin, the maximum perceived-unperceived ERP difference was computed. Errors bars, SEM.

Finally, we investigated the link between the prepulse α phase and amplitude, and the postpulse evoked activity. For each participant, we sorted the low-α and high-α amplitude trials in nine bins, as previously described, separately for electrodes PO3 and AFz. For each phase bin and α-amplitude condition, the maximum perceived-unperceived ERP difference was computed on a selected time-window of interest (see Materials and Methods; Fig. 3, gray shaded areas). A two-way repeated-measures ANOVA on electrode PO3 (Fig. 6*A*) revealed a significant main effect of the phase (*F*_(1,8)_ = 2.338, *p* = 0.0286, η^2^ = 22.62, SS = 430.66) and α-amplitude (*F*_(1,8)_ = 13.623, *p* = 0.0061, η^2^ = 63, SS = 137.62; this is coherent with the selection of the ERP time window of interest and will not be further interpreted; see Materials and Methods), but no significant interaction (*F*_(1,8)_ = 0.377, *p* = 0.9289, SS = 61.76). The two-way repeated-measures ANOVA on electrode AFz (Fig. 6*C*) showed a significant main effect of the α-amplitude (*F*_(1,8)_ = 12.749, *p* = 0.0073, η^2^ = 61.44, SS = 102.97; this is coherent with the selection of the ERP time window of interest and will not be further interpreted; see Materials and Methods), no significant effect of the phase (*F*_(1,8)_ = 0.393, *p* = 0.9206, η^2^ = 4.68, SS = 161.37), and no interaction (*F*_(1,8)_ = 0.376, *p* = 0.9297, SS = 158.75). In other words, for both low-α and high-α amplitude, the phase of prepulse spontaneous α oscillations predicts the ERP difference exclusively for the occipital electrode PO3. Specifically, we observed a higher ERP difference at –π/4, i.e., around the nonoptimal phase for phosphene perception (see Fig. 5*A*). This effect seems to come from an increased ERP in perceived-phosphene trials specifically. Indeed, we extracted the peak of the ERP for low-α and high-α amplitude trials, at –π/4 and π/2 phases of the α cycle, corresponding, respectively, to the maximum and the minimum ERP difference observed (see Fig. 5*A*), separately for perceived-phosphene and unperceived-ERPs, for the nine participants (Fig. 6*B*). We implemented two linear mixed effects models, one for each α-amplitude condition. In each model, we entered as fixed effects the phase (–π/4, π/2), the phosphene condition (perceived, unperceived), as well as their interaction. As random effect, we had participants’ intercepts and slopes for the effect of phase and phosphene condition. We observed a significant effect of the phosphene condition for both low-α (*t*_(32)_ = −3.1, *p* = 0.004, estimate = −12.201 ± 3.935, SE) and high-α (*t*_(32)_ = −3.252, *p* = 0.003, estimate = −12.188 ± 3.748, SE) amplitude conditions, a significant effect of the phase for low-α (*t*_(32)_ = −2.551, *p* = 0.0157, estimate = −10.161 ± 3.983, SE) and high-α (*t*_(32)_ = −2.313, *p* = 0.0273, estimate = −8.833 ± 3.82, SE) amplitude conditions, and a significant interaction between the phosphene condition and the phase for both low-α (*t*_(32)_ = 2.468, *p* = 0.0191, estimate = 6.142 ± 2.489, SE) and high-α (*t*_(32)_ = 2.237, *p* = 0.033, estimate = 5.292 ± 2.369, SE) amplitude conditions. A *post hoc* analysis showed that the ERP difference at –π/4 for perceived-phosphene trials was significantly higher compared with unperceived-phosphene trials at –π/4 for both low-α (one-tailed *t* test, *p* = 0.0265, Cohen’s *d* = 0.809, CI = [1.09; infinity]) and high-α (*p* = 0.0074, Cohen’s *d* = 1.069, CI = [2.753; infinity]) amplitude conditions, and compared with unperceived-phosphene trials at π/2 for both low-α (*p* = 0.0254, Cohen’s *d* = 0.477, CI = [0.747; infinity]) and high-α (*p* = 0.0253, Cohen’s *d* = 0.737, CI = [0.984; infinity]) amplitude conditions. Thus, around –π/4 for both low-α and high-α amplitude, we observed a low percentage of perceived-phosphene (Fig. 5*A*) associated with a high ERP difference when the phosphene is perceived (Fig. 6*A*).

## Discussion

In this study, we tested the two clear predictions of the pulsed inhibition theory (Klimesch et al., 2007; Jensen and Mazaheri, 2010; Mathewson et al., 2011): (1) high-α amplitude induces cortical inhibition at specific phases of the α cycle, leading to periodic perceptual performance; while (2) low-α amplitude is less susceptible to phasic (periodic) pulsed inhibition, leading to overall higher perceptual performance. Cortical excitability was assessed by both phosphene detection and postpulse evoked EEG activity. We showed that the prepulse phase of spontaneous α oscillations (∼10 Hz) modulates the probability to perceive a phosphene (with a nonoptimal phase between –π/2 and –π/4). This phase effect was stronger for high-α amplitude trials. Moreover, the prepulse nonoptimal phase leads to an increase in postpulse evoked activity (ERP), in phosphene-perceived trials specifically. Together, our results provide strong evidence in favor of the pulsed inhibition theory by establishing a causal link between the amplitude and the phase of spontaneous α oscillations, cortical excitability, and subsequent perceptual performance.

### α Phase-amplitude tradeoffs on perception

As previously described in the literature, we found that the phase of spontaneous oscillations in the α frequency range predicts whether a near-threshold stimulus would be successfully perceived (Busch et al., 2009; Mathewson et al., 2009; Dugué et al., 2011a; Samaha et al., 2015, 2017). The use of TMS to induce phosphene perception rather than an external stimulation allows for direct access to the absolute phase of spontaneous oscillations. We found that a phase between –π/2 and –π/4 was associated with inhibitory moments leading to lower perceptual performance while the opposite one (π/2) was optimal for perception. Critically, as predicted by the pulsed inhibition theory, our results are in line with some previous studies showing that the phase of spontaneous α oscillations better predicts perceptual performance for high than for low-α amplitude (Mathewson et al., 2009; Ng et al., 2012; Ai and Ro, 2014; Bonnefond and Jensen, 2015; Herrmann et al., 2016; Spitzer et al., 2016; Kizuk and Mathewson, 2017; Alexander et al., 2020) but not others (Busch and VanRullen, 2010; Zoefel and Heil, 2013; Milton and Pleydell-Pearce, 2016; Harris et al., 2018; Madsen et al., 2019). Other studies investigated the specific case in which a high-α amplitude condition is compared with the actual absence of α oscillations (Schaworonkow et al., 2018, 2019; Stefanou et al., 2018; Zrenner et al., 2018; Bergmann et al., 2019). They found periodic functional inhibition induced by μ oscillations (α oscillations recorded in the motor cortex) in the high-α amplitude condition (see next paragraph for more details). Here, we compared high-α amplitude trials to trials in which α oscillations were present but with a lower amplitude, and found a phase effect in both α-amplitude conditions, but strongest when α amplitude is high.

### α Phase-amplitude tradeoffs on cortical excitability

We observed that the phase and the amplitude of spontaneous α oscillations influence cortical excitability, only when there is subsequent perception. Indeed, a phase between –π/2 and –π/4 led to higher ERP exclusively for phosphene perception trials. Interestingly, this phase was also associated with lower perceptual performance. The nonoptimal phase of the α oscillations (between –π/2 and –π/4) tends to create periodic inhibitory cortical states favoring the absence of phosphene perception, which leads to a greater ERP response when a phosphene is in fact perceived. It is important to notice that the paradigm developed by Dugué et al. (2011a) was designed to specifically investigate the role of the phase of α oscillations (and not the phase-amplitude tradeoffs). However, the results are compelling and in line with other studies observing similar effects (Bonnefond and Jensen, 2015; Hussain et al., 2019; and others comparing high-α amplitude to the absence of α oscillations: Schaworonkow et al., 2018, 2019; Stefanou et al., 2018; Zrenner et al., 2018; Bergmann et al., 2019). In the motor modality, they used single-pulse TMS over the motor cortex to induce MEPs allowing to estimate corticospinal excitability. They found an increase in corticospinal excitability and the subsequent MEP for high-μ amplitude oscillations (i.e., α oscillations observed in somatosensory and motor areas) and for specific μ phases (Schaworonkow et al., 2018, 2019; Stefanou et al., 2018; Zrenner et al., 2018; Bergmann et al., 2019; Hussain et al., 2019). Bonnefond and Jensen (2015) alternatively analyzed the power of high γ oscillations (80–120 Hz) considered to reflects neuronal firing (Ray et al., 2008). They showed that γ power was weaker at the trough of high-α amplitude oscillations (Bonnefond and Jensen, 2015). As predicted by the pulsed inhibition theory, high-α amplitude modulates cortical and corticospinal excitability periodically. Interestingly, although the pulsed inhibition theory (Jensen and Mazaheri, 2010) originally proposed asymmetrical pulsed inhibition (i.e., inhibition at one particular phase and no inhibition at the opposite one), Bergmann et al. (2019) argued in favor of asymmetrical pulsed facilitation. Indeed, they assessed the role of the GABAergic system, considered the main source of inhibition in the brain (Ribak and Yan, 2000), on the amplitude and phase of μ oscillations, and did not observe any relation. The symmetry/asymmetry hypothesis was not explicitly assessed in the present study. Further investigation is thus necessary to disentangle the three possibilities: (1) symmetric pulsed inhibition and facilitation; (2) asymmetrical pulsed inhibition; or (3) asymmetrical pulsed facilitation.

### α, A top-down process?

Our results show a potential functional link between the occipital and the frontal lobes. Several authors have proposed that α carries feedback information (van Kerkoerle et al., 2014; Michalareas et al., 2016) and that the amplitude of occipital α oscillations is modulated by top-down connections from frontoparietal regions (Klimesch et al., 2007; Mathewson et al., 2011). Here, we can speculate that the frontal region plays a role in the emergence of inhibitory and excitatory moments in occipital cortex. Specifically, their top-down influence on the amplitude of α oscillations would enhance or reduce locally the effect of the phase of occipital α oscillations on perceptual performance, thus explaining that the link between the phase and the amplitude of α oscillations and cortical excitability (ERP) was only present in the occipital ROI. In addition, the previous study from which the data originate (Dugué et al., 2011a) shows that the time at which the phase predicted the perceptual outcome differed by nearly one-half α-cycle between the occipital (−77 ms) and the frontal (−40 ms) ROI. This difference may reflect the delay for neural information to be transferred from one brain region to the other, consistent with previous observations of an α phase difference between occipital and frontal regions during visual perception (Burkitt et al., 2000; Patten et al., 2012; Alamia and VanRullen, 2019; Pang et al., 2020; Tsoneva et al., 2021). Further studies are warranted to investigate the functional interplay between the frontal and occipital cortex in the context of the pulsed inhibition theory.

In conclusion, our study provides strong causal evidence in favor of tradeoffs between the phase and the amplitude of α oscillations to create periodic inhibitory moments leading to rhythms in perception. As predicted by the pulsed inhibition theory, the effect of the phase of spontaneous α oscillations on perception increases for larger α amplitude.

## Synthesis

Reviewing Editor: Nicholas J. Priebe, University of Texas at Austin Decisions are customarily a result of the Reviewing Editor and the peer reviewers coming together and discussing their recommendations until a consensus is reached. When revisions are invited, a fact-based synthesis statement explaining their decision and outlining what is needed to prepare a revision will be listed below. The following reviewer(s) agreed to reveal their identity: Til Bergmann.

SYNOPSIS

Two reviewers evaluated the manuscript “Alpha phase-amplitude tradeoffs predict visual perception” and both reviewers agreed that the manuscript is an important extension of a previous study demonstrating the impact of alpha phase amplitude on evoked responses by TMS. The reviewers did raise important issues with the manuscript, however. While the authors frame their analyses as a test of the pulsed inhibition hypothesis, the reviewers disagreed with the authors about the predictions of this hypothesis, and how it relates with the data presented. Further, the reviewers felt that there were major issues related to the identification of alpha, particularly for the low power epochs which require additional analysis. Finally, it was noted the simulations and data were not always aligned. We include detailed suggestions which you should find useful in revising your manuscript, if you decide to resubmit it to eNeuro or to another journal.

----------

REVIEWER 1

ADVANCES THE FIELD

New information on how alpha amplitude modulated the effect of alpha phase on vidual cortex excitability.

COMMENTS

Fakche et al., reanalyzed data from a previous TMS-EEG study (Dugué et al., J Neurosci 2011) to evaluate the impact of alpha amplitude on the earlier described effect of alpha phase on TMS-induced phosphene perception likelihood (as a measure of visual cortex excitability) and ERPs. Using a series of elegant analyses (most of them following the rationale of their previous publication), they showed that the phase effect is more pronounced for high- than low-power trials (more widespread across time and frequency domains as well as having a larger and more plausible topographical distribution), even though the two power conditions were not directly compared statistically for all measures.

This paper is an interesting extension of the previous Dugué et al., J Neurosci 2011 publication, as it post-hoc investigates how pre-stimulus alpha power interacts with alpha phase to predict visual cortex excitability (phosphene likelihood), which had not been explicitly addressed for visual cortex alpha so far. As such, this paper deserves publication, given that the methods are largely sound, the results well presented, and the paper well written. Regarding the authors’ interpretation of the findings in light of the Pulsed Inhibition Hypothesis (Jensen & Mazaheri, 2010), however, I do think the interpretation must be rephrased a bit (see specific comments below).

1. The authors state (eg in lines 552-557): “In this study, we tested the two clear predictions of the Pulsed Inhibition theory (Jensen and Mazaheri, 2010; Klimesch et al., 2007; Mathewson et al., 2011): (1) high alpha amplitude induces cortical inhibition at specific phases of the alpha cycle, and excitation at opposite ones, leading to periodic perceptual performance; and (2) low alpha amplitude induces overall higher neuronal excitability, less susceptible to phasic (periodic) pulsed inhibition, and lead to overall higher perceptual performance”.

However, the pulsed inhibition hypothesis does not claim inhibition and excitation at opposite phase angles, but based on the assumed asymmetry of the alpha oscillation, it assumes in particular a pulsed inhibition at one particular phase angle and no inhibition or facilitation (relative to the absence of the oscillation) on the opposite phase angle. An important question is thus whether the data actually supports asymmetrical pulsed inhibition, asymmetrical pulsed facilitation, or symmetric inhibition and facilitation (for the rationale see Bergmann et al. J Neurosci 2019). Therefore, it should be tested whether the phosphene likelihood differs particularly between low and high amplitude trials for one of the phase angles but not for the other, and if so whether it is particularly inhibited or facilitated relative to the low power trials.

2. The authors made a lot of efforts (with control analyses and simulations) to rule out that their findings can be explained by a methodological confound, i.e., the mere fact that phase estimation is less accurate for low power trials, and this is very much appreciated. However, I think an even more fundamental point still requires discussion, namely that, in principle, low power may also reflect the actual absence of an actual alpha oscillation at the neurophysiological level, i.e., a desynchronized state, and that the power revealed by an FFT (or wavelet or band-pass filtered signal, etc.) simply reflects the aperiodic 1/f background noise. It is thus generally questionable whether the estimation of phase in a low power signal is neurophysiologically meaningful. This can be elucidated by providing power spectra for all power bins and determine the presence of an alpha frequency peak, or even separating the low power bin into trials with and without an alpha frequency peak in the power spectrum on a single trial basis.

3. In relation to comment #2: I understand that binning of single-trial power values is necessary for a factorial design, and that only a limited number of bins are possible to ensure sufficient trial numbers per bin. However, the specific binning into 2, 3, 4, or more bins, may have a fundamental impact on the results, and the choice of 3 bins (and eventually only 2 bins) does not seem to be theoretically motivated. Do the results look similar when using more bins? When exploiting the entire variance of the continuous alpha power values, what does a logistic regression reveal using alpha power as predictor and phosphene perception as binary criterion?

4. Line 72: (and similar in line 593): “Other studies found a causal phase effect of spontaneous alpha oscillations on cortico-spinal excitability and the associated MEP for high-alpha amplitude, but without testing for low-alpha amplitude (Bergmann et al., 2019; Schaworonkow et al., 2019, 2018; Stefanou et al., 2018; Zrenner et al., 2018).” In fact, Bergmann et al., 2019 did explicitly test a low-amplitude condition to compare with the different phase angles of the high amplitude condition. While this did indeed not investigate a differential effect of phase angle for the low-amplitude condition itself, it did so for the reason addressed in comment #2, namely that no reliable physiological oscillation was present at the targeted lower end of the individual alpha power distribution, but it did allow to test the explicit scenarios outlined in comment #1. Maybe the implications of choosing a specific bin of “low alpha power” should be discussed explicitly, as depending on their borders those bins may or may not contain actual alpha oscillations and thus different questions can be addressed by comparing them to high amplitude alpha.

5. Line 338: “The analysis was replicated on low-alpha amplitude trials (Figure 1, bottom raw). The overall strength of the effect was less important (remained significant after FDR correction, FDR = 0.01, corresponding to a Z-score threshold of 4.26, a p-value threshold of 2.08 x 10 -5 , and a Cohen’s d approaching infinity), and showed a less informative topography of the effect.” It is unclear what “overall strength of the effect was less important” means, and the phase-opposition effect does not seem to be directly compared between low- and high-amplitude trials, which it should be to make the claim that they differ (especially since the statistical values are very similar for the two separate analyses).

6. Line 560: “This phase effect appears more robust across time and across frequencies for high-alpha amplitude trials.” and further Line 618: “Conclusion. Our study provides strong causal evidence in favor of tradeoffs between the phase and the amplitude of alpha oscillations to create periodic inhibitory moments leading to rhythms in perception, and validate the predictions made by the Pulsed Inhibition theory.”

The conclusion remains a bit vague. Are the authors concluding based on the available results that the phase effect is stronger for high- than low-amplitude trials (is there a direct statistical comparison backing that up) OR that it did not differ, but is at least present for both low- and high-amplitude trials? What would be predicted by the pulsed inhibition hypothesis, and which aspect of the pulsed inhibition hypothesis is actually tested here?

7. When analyzing the post-TMS ERPs for different phases of the pre-TMS alpha rhythm, the problem is neglected that the ongoing alpha oscillation will continue into the post-TMS period (if not completely phase reset by TMS, for which there is no evidence), and the phosphene related ERP will thus overlay (superimpose or interact with) different phases of the still ongoing alpha oscillation. The ERP amplitude may thus be confounded by the phase of the alpha oscillation at the time of stimulation (and thus 400 ms later). Unless unstimulated trials of comparable amplitude and phase are used to remove that impact (by subtraction from the stimulated trials), any difference in the post-TMS ERPs cannot be easily interpreted.

Minor

8. Is the power spectrum of the pre-TMS ERP, shown in the Figure 3, corrected for the 1/f aperiodic activity? It looks like the high-alpha amplitude condition also has a higher 1/f component than the low-alpha amplitude, and the power difference between the conditions may disappear (be non-significant anymore) when 1/f correction would be applied. In Line 253, a 1/f component is mentioned, but it remains unclear whether a correction was performed and if so how exactly.

9. Line 107: The orientation of the TMS coil and the resulting direction of current flow in the brain tissue (for the second, most relevant half wave of the biphasic pulse) should be stated explicitly.

10. Line 124: Which hand was used to press the buttons (or both)? This may affect alpha oscillations in the respective contralateral sensorimotor cortex and affect the occipital alpha oscillation analysis.

11. Line 154: Why averaging across all electrodes (alpha power fluctuations in different cortical regions can even be anticorrelated)?

12. Line 156: Why only until -50 ms?

13. Line 156: Why up to 17 Hz (which is clearly not alpha anymore)?

14. On several occasions it says “[authors’ previous publication]” instead of citing the respective paper. This should be corrected.

15. Line 161 to 185: the process of calculating a derived measure of phase angle i.e., phase opposition sums (POS) is described, but the details on basic phase estimation are missing. Similarly, in line 153-160: the details to sort trials based on alpha amplitude are given but the details of the method to measure amplitude is not reported.

16. Please report how many trials are left per condition for each analyses.

17. Please report how many bad channels were removed.

18. Please report the sampling rate and filter properties of the original EEG recording.

19. A Figure showing the task could be shown (even though described in the original publication).

----------

REVIEWER 2

ADVANCES IN THE FIELD

The analyses are mostly confirmatory but do provide additional evidence demonstrating the effects of alpha amplitude and phase on perception.

COMMENTS

This manuscript describes the results of new analyses conducted on previously collected and already published data examining how alpha amplitude and phase affect TMS-induced phosphene perception. The authors report that at high but not low levels of alpha amplitude, the phase of alpha oscillations affects phosphene perception.

Overall, this is an interesting manuscript that provides further support for the pulsed inhibition theory, which essentially proposes that alpha oscillations phasically inhibit perception. However, whether the simulation and new analyses on phase-amplitude tradeoffs is novel enough for publication in a journal like eNeuro is questionable. Even though these results are more confirmatory than novel, perhaps they could add to the growing literature demonstrating the importance of alpha oscillatory phase on perception provided adequate revisions are made.

Currently, the manuscript is rather diffucult to follow and some of the analyses are seemingly arbitrary. Most notably, it is unclear why instantaneous phase was computed at -77 and -40 ms for the occipital and frontal ROIs, which would affect and shift the phase calculations by nearly a half to three-quarters of a cycle with respect to TMS onset. The claims that specific phase angles produce different effects are therefore very unconvincing. In fact, if the phase angles were computed at TMS onset, a very different pattern of results would likely emerge. Therefore, much further justification for selecting those time intervals is required.

Relatedly, other aspects of the statistical analyses are unclear, perhaps because of repeated references to the undisclosed authors’ previous publication.

Other comments:

The results from the low alpha amplitude oscillations in the simulated data seem to contradict the actual data. Some discussion of why this is the case may improve the flow of the manuuscript.

It is unclear what is meant by, “double pulses (40 ms-interval) were administered to monitor the validity of participants’ responses”. How would double pulses validate reports and were participants asked to report how many phosphenes they perceived?

The data from perceived and unperceived trials should be plotted separately in Figure 2.

There are several grammatical and typographical errors throughout the manuscript (e.g., line 45: “studies shown"; line 340: what is meant by less important?; line 584: Which phase? Perhaps phase range or phase window would be clearer).
