# Supplementary figures and images for: α Phase-Amplitude Tradeoffs Predict Visual Perception
Source: eNeuro. 2022 Feb 22;9(1):ENEURO.0244-21.2022. doi: 10.1523/ENEURO.0244-21.2022 (PMC8868024; doi:10.1523/ENEURO.0244-21.2022)

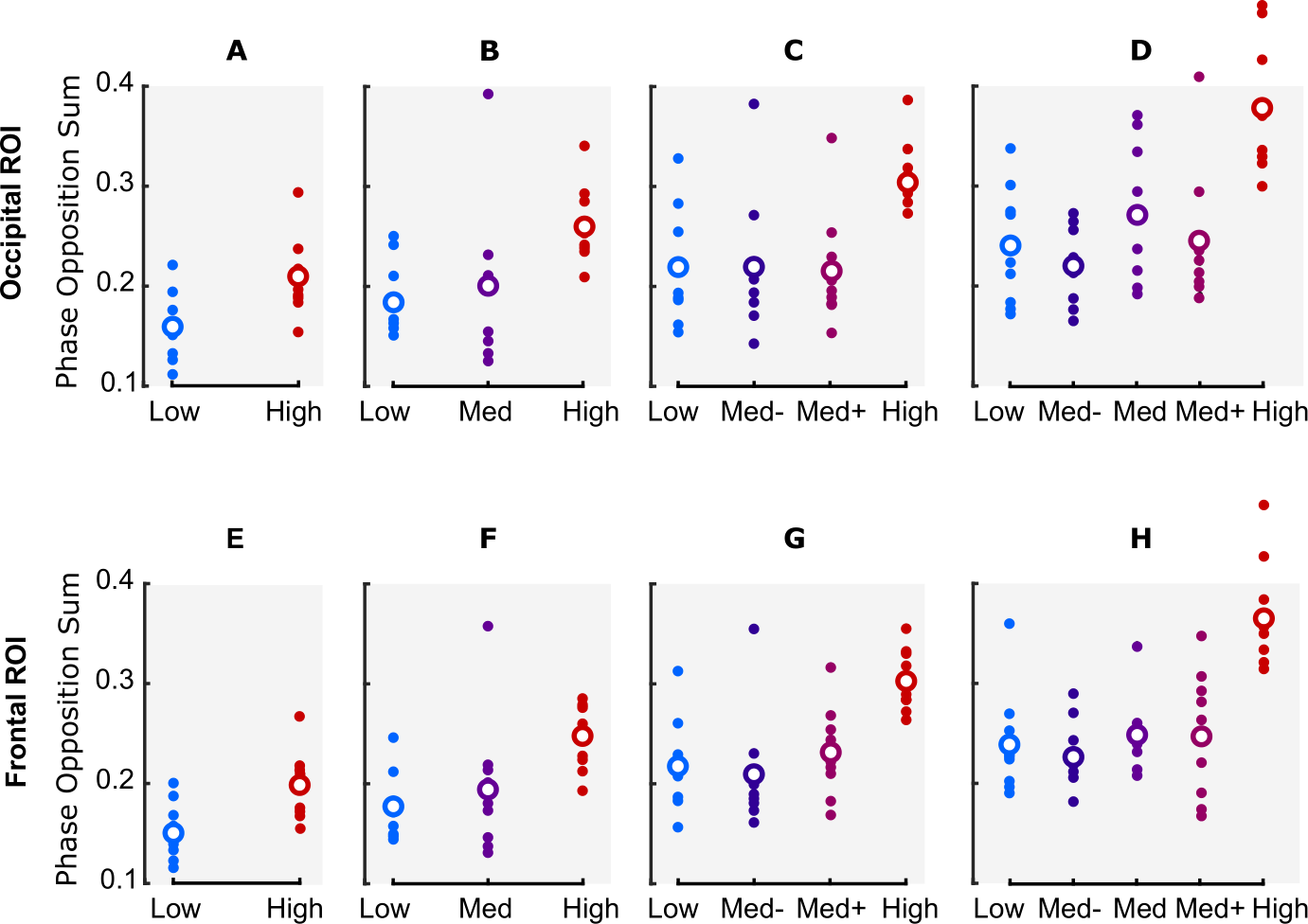

Supplement: Extended Data Figure 2-1 — The phase effect on phosphene perception is higher for high-α compared to low-α amplitude trials. POS computed for several binning versions of α amplitude trials, at 10.7 Hz and (A–D) at –77 ms prepulse, and averaged across electrodes within the occipital ROI, (E–H) at –40 ms prepulse, and averaged across the electrodes within the frontal ROI. Dots, POS for individual participants. Circles, POS averaged across the nine participants. All following analyses were performed on the three-bin condition (B, F), i.e., trials were binned in low-α, medium-α (med), and high-α amplitude trials, discarding the medium-α amplitude bin. Download Figure 2-1, TIF file. [file enu-eN-NWR-0244-21-s02.tif]

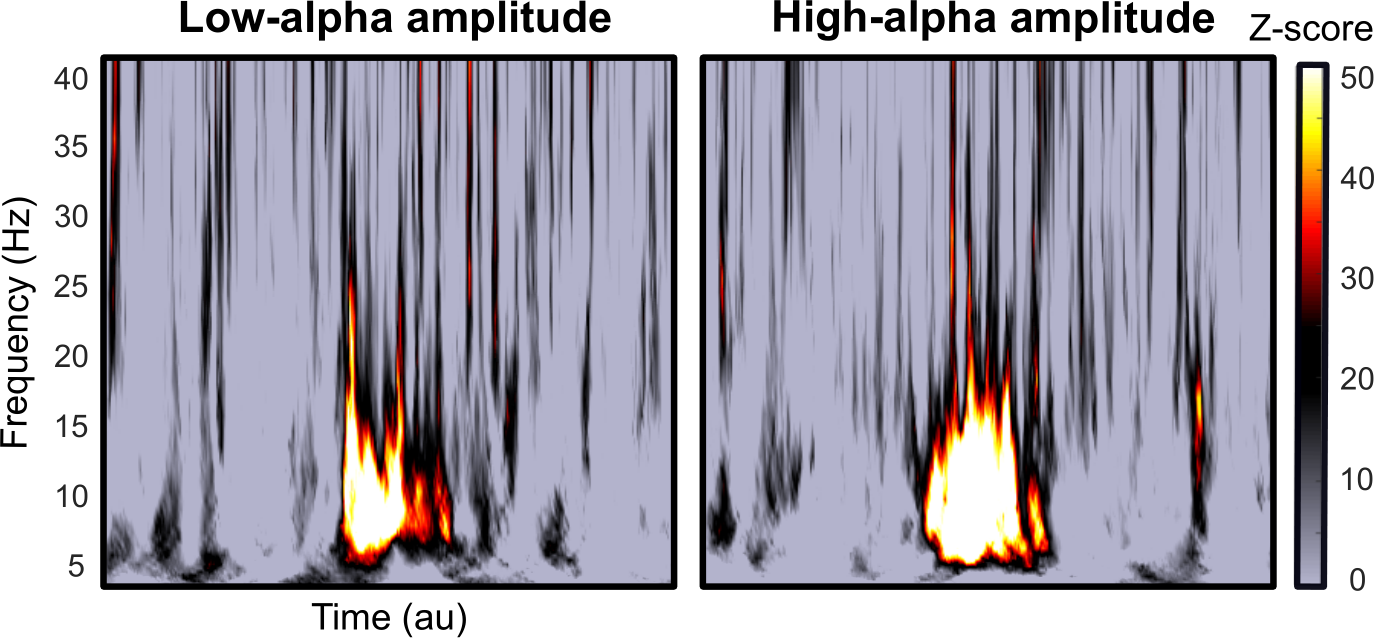

Supplement: Extended Data Figure 2-2 — Both low-α and high-α amplitude oscillations simulated datasets show a similar phase effect on perception. Left panel, Phase-opposition computed on simulated low-α amplitude trials. Right panel, Simulated high-α amplitude trials. Z-scores maps of phase-opposition between perceived-phosphene and unperceived-phosphene conditions. Colormap, Z-scores. Between low-α and high-α amplitude simulated trials, there is a comparable phase-opposition between perceived-phosphene and unperceived-phosphene conditions, from 5 to 18 Hz. Download Figure 2-2, TIF file. [file enu-eN-NWR-0244-21-s03.tif]

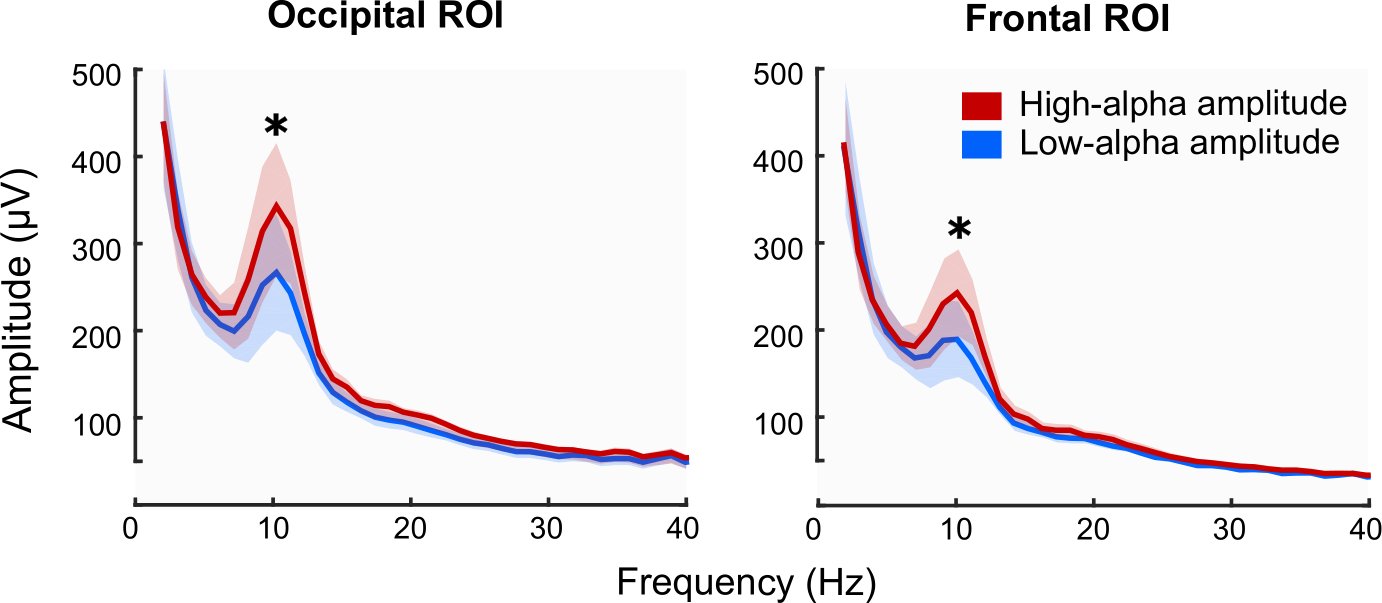

Supplement: Extended Data Figure 2-3 — Prepulse oscillatory activity in the α frequency band in both low-α and high-α amplitude conditions. Amplitude spectra computed on the EEG time-series from –600 to –1 ms relative to pulse onset, for the occipital ROI (left panel) and the frontal (right panel) ROI. Red color, high-α amplitude condition; blue color, low-α amplitude condition. Colored solid lines, amplitude spectra averaged across the nine participants between 2 and 40 Hz. Colored shaded areas, SEM. *, significant difference at 10.24 Hz between low-α and high-α amplitude conditions. Download Figure 2-3, TIF file. [file enu-eN-NWR-0244-21-s04.tif]

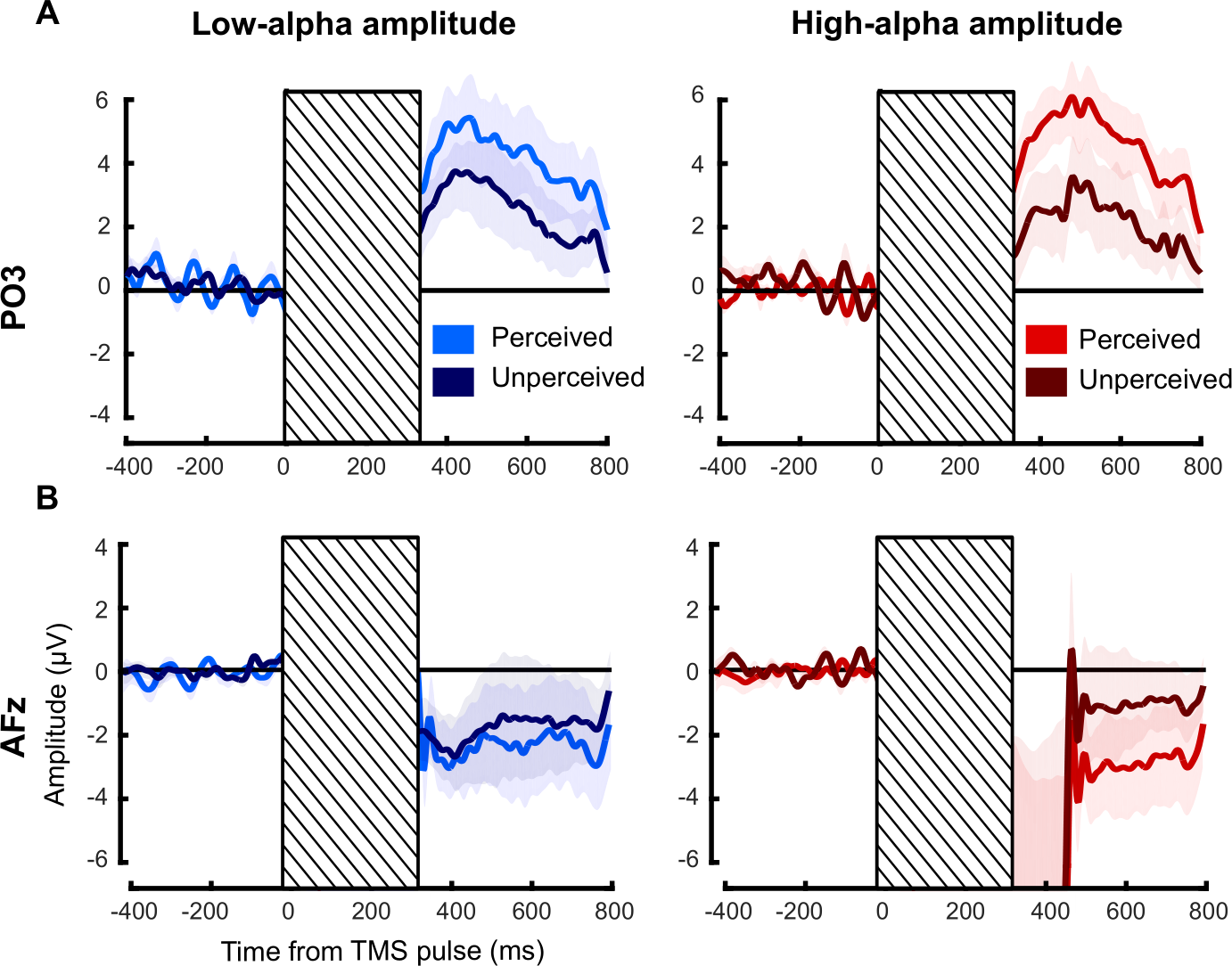

Supplement: Extended Data Figure 3-1 — ERPs for perceived-phosphene and unperceived-phosphene trials. A, ERPs at electrode PO3 for perceived-phosphene and unperceived-phosphene trials averaged across the nine participants. B, ERPs at electrode AFz. Red, high-α amplitude condition; blue, low-α amplitude condition. Light colors, perceived-phosphene condition; dark colors, unperceived-phosphene condition. Colored shaded areas, SEM. Striped area, mask the TMS-induced artifact. Download Figure 3-1, TIF file. [file enu-eN-NWR-0244-21-s05.tif]
